# Supplementary material for: Effect of tailoring anticoagulant treatment duration by applying a recurrence risk prediction model in patients with venous thromboembolism compared to usual care: A randomized controlled trial
Source: PLoS Med. 2020 Jun 26;17(6):e1003142. doi: 10.1371/journal.pmed.1003142 (PMC7319277; doi:10.1371/journal.pmed.1003142)
Supplement: S1 Table — DVT, deep vein thrombosis; ITT, intention to treat; PE, pulmonary embolism; PP, per protocol. (DOCX) [file pmed.1003142.s002.docx]

**S1 Table: Comparison of patients included in the ITT analysis and patients with non-adherence as included in the PP analysis**

|  | **Total index ITT**  **(N=441)** | **Total control ITT (N=442)** | **All patients in PP**  **(N=779)** | **Patients excluded from PP**  **(N=104)** |
| --- | --- | --- | --- | --- |
| Male sex – no. (%) | 253 (57%) | 254 (58%) | 429 (55%) | 78 (75%) |
| Age – year, mean (SD) | 55 years (14) | 55 years (14) | 55 years (14) | 56 years (15) |
| Diabetes mellitus – no. (%) | 24 (5%) | 30 (7%) | 49 (6%) | 5 (5%) |
| Previous cardiovascular disease – no. (%) | 26 (6%) | 31 (7%) | 46 (6%) | 3 (3%) |
| Index event – no. (%) |  |  |  |  |
| *Isolated (distal or proximal) DVT* | 218 (49%) | 216 (49%) | 393 (50%) | 41 (39%) |
| *PE (with or without DVT)* | 223 (51%) | 226 (51%) | 386 (50%) | 63 (61%) |
| Hormonal therapy – no. (%) | 90 (20%) | 94 (21%) | 175 (22%) | 9 (9%) |
| Known thrombophilia – no. (%) | 44 (10%) | 36 (8%) | 69 (9%) | 11 (11%) |
| History of DVT or PE – no. (%) | 21 (5%) | 21 (5%) | 39 (5%) | 3 (3%) |
| Initial treatment duration – no. (%) |  |  |  |  |
| *3 to 6 months* | 425 (96%) | 432 (98%) | 756 (97%) | 102 (98%) |
| *6 to < 12 months* | 16 (4%) | 10 (2%) | 23 (3%) | 2 (2%) |
