# Supplementary material for: Effect of tailoring anticoagulant treatment duration by applying a recurrence risk prediction model in patients with venous thromboembolism compared to usual care: A randomized controlled trial
Source: PLoS Med. 2020 Jun 26;17(6):e1003142. doi: 10.1371/journal.pmed.1003142 (PMC7319277; doi:10.1371/journal.pmed.1003142)
Supplement: S2 Table — All DVT index events were proximal. $D-dimer concentration as obtained at the first time the VPM was obtained (VPM1). #Depicts the days after VPM 1 is performed (see Fig 1). DVT, deep venous thrombosis; F, female; M, male; PE, pulmonary embolism. (DOCX) [file pmed.1003142.s003.docx]

**S2 Table: Description of 5 patients with interval recurrent venous thrombo-embolic event**

| **Patient no.** | **Gender (M/F)** | **Age (Yrs)** | **D-dimer, mcg/L ^$^** | **Index event** | **Days stopped ^#^** | **VPM 1 risk, %/year** |
| --- | --- | --- | --- | --- | --- | --- |
| 1 | M | 65 | 560 | DVT | 16 | 6.0% |
| 2 | F | 46 | 370 | DVT | 13 | 3.0% |
| 3 | M | 80 | 431 | DVT | 19 | 5.5% |
| 4 | F | 75 | 297 | PE | 14 | 3.4% |
| 5 | M | 55 | <190 | PE | 28 | <5.2% |
