# Supplementary material for: Effect of tailoring anticoagulant treatment duration by applying a recurrence risk prediction model in patients with venous thromboembolism compared to usual care: A randomized controlled trial
Source: PLoS Med. 2020 Jun 26;17(6):e1003142. doi: 10.1371/journal.pmed.1003142 (PMC7319277; doi:10.1371/journal.pmed.1003142)
Supplement: S3 Table — #These estimates come from the following line of reasoning: We know that the proportion of patients categorized in the following risk strata of annualized VTE recurrence risk of 0% to 2%, >2% to 4%, >4% to 5%, and >5% are 4.9%, 35.1%, 17.1%, and 42.9%, thus 49, 351, 171, and 429 patients in a hypothetical cohort of 1,000 patients, respectively. Based upon our VISTA data, we also know the observed annualized rates of recurrent VTE in these risk strata if anticoagulant treatment is withheld, i.e., 0.0%, 2.5%, 6.7%, and 14.4%, respectively. We know from the NOAC extension trials that these risks of recurrent VTE can be lowered with NOAC treatment, with an observed hazard ratio in these trials of about 0.25, thus yielding annualized rates of recurrent VTE, if managed with prolonged NOAC treatment, of 0.0%, 0.63%, 1.68%, and 3.6%, respectively. Now, let’s assume the annualized rates of recurrent VTE to be constant over a 5-year period. This is a conservative assumption, as we know that the rates of recurrent VTE are highest in the first year after the initial event and typically a little bit lower in the subsequent years. Using these data, we can estimate the number of VTE events over a 5-year period in our hypothetical cohort of 1,000 VTE patients for the following scenarios: (i) prolong treatment in all patients, (ii) prolong only if the estimated risk of recurrence is >2%, (iii) prolong only if the estimated risk of recurrence is >4%, (iv) prolong only if the estimated risk of recurrence is >5%, or (v) do not prolong anticoagulant treatment in any patients. In fact, the number of recurrent VTE events for these scenarios would be 103, 103, 135, 178, and 410 events, or, compared to prolonging treatment in all patients (first scenario), 0, 0, 33, 76, and 308 additional events, respectively. Similarly, we can estimate the number of major bleeding events induced in these different scenarios. Unfortunately, the annualized rate of major bleeding in VTE patients on prolonged [file pmed.1003142.s004.docx]

**S3 Table: Clinical outcomes in a hypothetical cohort of 1000 VTE patients, followed-up for 5 years**

| **Treatment strategy** | **Number of treated patients #** | **Recurrent VTE #** |  | **Major bleeding** |  |
| --- | --- | --- | --- | --- | --- |
|  |  | *number* | *additional VTE* |  | *number* |
| **Treat all patients** | 1000 | 90 | 0 | Rate 0.5% per year | 25 |
|  |  |  |  | Rate 1.0% per year | 50 |
|  |  |  |  | Rate 1.5% per year | 75 |
| **Treat only >2% risk/year** | 940 | 90 | 0 | Rate 0.5% per year | 24 |
|  |  |  |  | Rate 1.0% per year | 47 |
|  |  |  |  | Rate 1.5% per year | 71 |
| **Treat only >4% risk/year** | 530 | 128 | 38 | Rate 0.5% per year | 13 |
|  |  |  |  | Rate 1.0% per year | 27 |
|  |  |  |  | Rate 1.5% per year | 40 |
| **Treat only >5% risk/year** | 340 | 177 | 87 | Rate 0.5% per year | 9 |
|  |  |  |  | Rate 1.0% per year | 17 |
|  |  |  |  | Rate 1.5% per year | 26 |
| **Treat no patients** | 0 | 361 | 270 | Rate 0.5% per year | 0 |
|  |  |  |  | Rate 1.0% per year | 0 |
|  |  |  |  | Rate 1.5% per year | 0 |
