# Supplementary material for: Effect of tailoring anticoagulant treatment duration by applying a recurrence risk prediction model in patients with venous thromboembolism compared to usual care: A randomized controlled trial
Source: PLoS Med. 2020 Jun 26;17(6):e1003142. doi: 10.1371/journal.pmed.1003142 (PMC7319277; doi:10.1371/journal.pmed.1003142)
Supplement: S1 Text — (PDF) [file pmed.1003142.s005.pdf]

Research Protocol

[redacted]

Efficacy of tailoring anticoagulant therapy by a VTE recurrence prediction model in patients with venous thrombo-embolism as compared to care-as-usual: the VISTA study.

Version 6.0

Latest revision: 20 april 2015

University Medical Center (UMC) Utrecht
Julius Center for Health Sciences and General Practice

Protocol ID:	ZonMW project number: 171002214
Short title:	The VISTA study
Version:	6.0
Date:	20 April 2015

Coordinating investigator: Prof. Dr. K.G.M. Moons – Clinical Epidemiologist
E-mail: k.g.m.moons@umcutrecht.nl
University Medical Center Utrecht
Julius Center for Health Sciences and Primary Care
PO Box 85500 3508 GA UTRECHT

Sponsor: ZonMW project number: 171002214
Unrestricted educational grant: Bayer Shering Pharma- Mijdrecht The Netherlands

Independent physician: Dr. F.H. Rutten- General Practitioner
Nieuwe Veenendaalseweg 51 a
3911 ME Rhenen
T: 0317- 650170

Laboratory sites:

Trombosedienst Saltro	Utrecht
Trombosedienst Amersfoort	Amersfoort
Trombosedienst Neder Veluwe	Ede
Trombosedienst INR	Nijmegen
Trombosedienst St. Jansdal	Harderwijk
Trombosedienst Isala Klinieken	Zwolle
Trombosedienst Medlon	Enschede
Trombosedienst Deventer Ziekenhuis	Deventer
Trombosedienst voor het Gooi	Hilversum
Trombosedienst STAR-MDC	Rotterdam

PROTOCOL SIGNATURE SHEET

Name	Signature	Date
For non-commercial research,<br>Head of Department:<br>Prof. Dr. K.G.M. Moons<br>Professor of clinical epidemiology		
Coordinating Investigator/Project leader/Principal Investigator:<br>Dr. R. Oudega<br>Project leader		

TABLE OF CONTENTS

1. INTRODUCTION AND RATIONALE 4
2. OBJECTIVES 5
3. STUDY DESIGN 6
4. STUDY POPULATION 8
4.1 Population (base) 8
4.2 Inclusion criteria 8
4.3 Exclusion criteria 8
4.4 Sample size calculation 8
5. TREATMENT OF SUBJECTS 9
5.1 Investigational product/treatment 9
5.2 Use of co-intervention (if applicable) 9
5.3 Escape medication (if applicable) 9
6. METHODS 10
6.1 Study parameters/endpoints 11
6.1.1 Main study parameter/endpoint 11
6.1.2 Secondary study parameters/endpoints (if applicable) 11
6.2 Randomisation, blinding and treatment allocation 11
6.3 Study procedures 12
6.4 Withdrawal of individual subjects 13
6.4.1 Specific criteria for withdrawal (if applicable) 13
6.5 Premature termination of the study 13
7. SAFETY REPORTING 13
7.1 Section 10 WMO event 13
7.2 Monitoring 13
7.3 Adverse and serious adverse events 13
7.4 Follow-up of adverse events 14
7.5 Data Safety Monitoring Board (DSMB) 15
8. STATISTICAL ANALYSIS 14
8.1 Descriptive statistics 14
8.2 Multivariate analysis 14
8.3 Interim analysis 14
9. ETHICAL CONSIDERATIONS 15
9.1 Regulation statement 15
9.2 Recruitment and consent 15
9.3 Compensation for injury 15
9.4 Outlining responsibilities during study 15
10. ADMINISTRATIVE ASPECTS AND PUBLICATION 17
10.1 Handling and storage of data and documents 17
10.2 Amendments 17
10.3 Annual progress report 17
10.4 End of study report 17
10.5 Public disclosure and publication policy 17
12. REFERENCES 18

SUMMARY

Rationale: Long-term treatment with vitamin K antagonists (VKA) in patients with venous thromboembolism (VTE) – this is deep vein thrombosis (DVT) and pulmonary embolism (PE) – aims at reduction of recurrent VTE while prevention of major bleedings (complication from VKA treatment) has to remain essential. There is limited evidence and thus much discussion and practice variation regarding optimal long-term duration of VKA treatment in different patient groups. (Inter) national guidelines advice individual evaluation for the risk-benefit ratio of long-term treatment. Recent studies, including a small RCT, suggest tailoring of treatment duration using various patient characteristics and D-dimer level measurement(s). The cost-effectiveness and efficacy of such tailored treatment strategies are unknown.

Objective: Quantify the efficacy and cost-effectiveness of tailoring the duration of VKA treatment with a formal prediction model using patient characteristics and repeated D-dimer testing in VTE patients, compared to treatment decisions by physicians based on clinical experience and own discretion ('care as usual').

Study design: Randomised pragmatic multi-center intervention study.

Study population: Patients with a (first) unprovoked VTE who are treated with VKA. Patients are recruited from Dutch Thrombosis Services.

Intervention: *Index*: Evaluating the risk-benefit ratio of long-term VKA treatment by a prediction model – combining patient characteristics and D-dimer testing. The prediction model is applied after an initial period of usually 6 months of VKA treatment. If the prediction model yields a low risk of VTE recurrence, treatment is ceased. If the model yields a high risk of VTE recurrence, treatment is continued or resumed for another 24 months. Treating physicians can overrule this decision if a high bleeding risk is anticipated during VKA treatment. *Control*: care as usual; this is conform physicians' discretion based on current guidelines.

Main study parameters/endpoints: *Primary:* recurrent VTE (proximal DVT and fatal or non-fatal PE) during 24 months of follow-up. *Safety and secondary outcomes*: major bleedings, quality of life and cost-effectiveness.

Nature and extent of the burden and risks associated with participation, benefit and group relatedness: Patients randomized to the intervention group undergo up to a maximum of two D-dimer tests (blood tests), thus also maximally two additional site visits (at their local Thrombosis Service). As a consequence of these test results (and other patient characteristics) patients can be treated for a longer period of time with VKA as compared to usual care. Although it is anticipated that this will result in fewer recurrent VTE events, patients may be at increased or similar risk of VTE and/ or bleeding as compared to patients randomized to the control group. Patients randomized to the control group will receive usual care. For comparison reasons D-dimer testing will also be performed after (usually) 6 months of VKA treatment and one month after discontinuation of VKA in these patients. During the follow-up period of two years the investigators will approach all patients three times (at 3, 12 and 24 months after the end of the initial VKA treatment). At each follow-up moment a few questions will be asked. Furthermore – as part of the cost-effectiveness analyses – patients are asked to fill in 'quality of life' questionnaires at baseline, the moment of intervention and prior to each follow-up contact.

1. INTRODUCTION AND RATIONALE

Treatment of venous thrombo-embolism (VTE) – this is deep vein thrombosis (DVT) and pulmonary embolism (PE) – with anticoagulant therapy, usually vitamin K antagonists (VKA), is highly effective in reducing morbidity and mortality.[1,2] It reduces mortality caused by PE from 30 to 8%.[3,4] The initial treatment of VTE consists of at least 5 days of heparin (unfractionated or low-molecular-weight) overlapped with 3-6 months VKA (acenocoumarol, phenprocoumon or warfarin). Long-term treatment after initial therapy reduces the risk of recurrent VTE by over 90%.[5,7] However, after VKA discontinuation there is considerable risk of VTE recurrence, about 9% after one year.[2,5] VTE recurrence has a case-fatality rate of 5%.[6] VTE recurrence is highest immediately after VKA therapy discontinuation, and stabilizes in the subsequent 9 months.[7] Long term treatment with VKA reduces the risk of VTE recurrence to 1% a year, but at a cost of 2% increasing risk of major bleedings.[8] Given the challenge to obtain (substantial) reduction in recurrent VTE risk versus increased risk of bleedings, there is much discussion about the optimal duration of VKA treatment in patients with VTE.[8,9] National and international guidelines on this topic suggest different strategies. The guideline from the 8th ACCP advices indefinite anticoagulant therapy for patients with a first unprovoked proximal DVT or PE and a low risk of bleeding.[9] The Dutch CBO consensus recommends for the same patients 6 months VKA therapy and indefinite when recurrence occurs.[5] However, there is limited evidence to ground these strategies. Caregivers and patients wrestle continuously with this question resulting in much uncertainty and widely varying durations of VKA therapy in daily practice.[10,11]

Observational studies show in patients with a first episode of unprovoked VTE, that a positive D-dimer test after VKA treatment indicates increased VTE recurrence risk [12-21,23]. This finding suggests that D-dimer testing can be used to determine in whom VKA prolongation seems beneficial. Hence, D-dimer guided VKA treatment strategy could minimize VTE recurrence (in whom longer VKA treatment is needed) and bleeding risk (in whom VKA treatment can be stopped). More recently, Eichinger and colleagues developed a prediction model that accurately predicts the risk of VTE recurrence in patients with a first episode of unprovoked VTE [22]. This prediction model was obtained by analysing several widely accepted factors related to VTE recurrence (Age, sex, location of VTE, BMI, factor V Leiden mutation, D-dimer levels and thrombin levels) in a cohort consisting of 929 VTE patients with a recurrence rate of 18.9% during a follow-up period of 43 months after VKA withdrawal. Only male sex, localisation (proximal DVT or PE) and elevated D-dimer levers were significantly associated with recurrence risk using a Cox proportional hazards model. Therefore, these factors were incorporated into the prediction model. The definition of a high recurrence risk if the risk exceeds 5% in 1 year, or 20% in 5 years, is based on widely accepted experts' consensus [24].

If for example – based on this model – the predicted VTE recurrence after stopping VKA treatment rate is high, patients may benefit from prolonged VKA treatment to prevent morbidity or even mortality that is associated with these VTE recurrences. If on the other hand the predicted VTE recurrence rate is low, patients can safely stop

VKA treatment as possible VKA treatment complications – including (major) bleedings – no longer outweigh the benefits of long-term VKA treatment in these patients.

Such a 'VTE recurrence prediction strategy' could increase the cost-effectiveness of VKA therapy in patients with VTE. Moreover, it gives physicians a more objective tool to tailor optimal duration of VKA therapy. This strategy of formal risk assessment has never been compared to care-as-usual in a randomized clinical trial (RCT). Only one small RCT exists on a D-dimer guided duration of VKA treatment. This RCT randomized VTE patients with high D-dimer levels at 3 months, to either stopping (control group) or continuing (index group) VKA treatment.[16] Patients with elevated D-dimer level at 3 months of treatment indeed benefit from prolonged VKA therapy: VTE recurrence over on average 18 months was 15.0% in the control group (with no bleedings) versus 2.9% (with 1% bleedings) in the index group. As it was the first study in this area, it only included a small group of patients with unprovoked VTE. Moreover, it only randomized patients with an elevated D-dimer level. Hence, it remains unclear whether an even more accurate prediction model that also includes other patient characteristics and D-dimer testing has an improved overall effectiveness compared to care-as-usual with duration of VKA treatment to the physicians' discretion, let alone whether it is cost-effective. Prompted by the promising results of this small trial, the less straightforward clinical guidelines resulting in the large uncertainty of physicians when to stop VKA treatment, the resulting practice variation, we propose this study of evaluating the existing guideline with a formal risk assessment.

2. OBJECTIVES

The objective is to quantify the cost-effectiveness and efficacy of formally applying the guideline evaluating the risk-benefit ratio of the duration of anticoagulant therapy (VKA treatment) by a previously developed VTE recurrence prediction model (including type of VTE, gender and D-Dimer testing [22]) in patients with a (first) episode of unprovoked VTE as compared to care-as-usual where the duration of VKA treatment is conform current guidelines and physicians' discretion. Unprovoked VTE is defined as a VTE event without surgery or immobilisation within 3 months prior to event, malignancy (latest treatment < 6 months prior to event), or pregnancy and puerperium up to 6 weeks postpartum.

The tailoring of the (dis)continuation of VKA treatment will be done usually after 6 months of initial VKA treatment. The effectiveness will be expressed by comparing the cumulative incidences in recurrent VTE and bleeding complications over 24 months of follow-up.

3. STUDY DESIGN

This is a pragmatic randomised multi-center trial in patients with a documented (first) episode of unprovoked DVT or PE. Unprovoked VTE is defined as a VTE event without surgery or immobilisation within 3 months prior to event, malignancy (latest treatment < 6 months prior to event), or pregnancy and puerperium up to 6 weeks postpartum.

After presentation, informed consent will be obtained. One month before the intervention, patients will be randomised to the index group (prediction model guided treatment of VKA, see flowchart below) or to the control group (care-as-usual, see flowchart below).The blocked randomisation will be performed within strata for centre and for type of VTE (DVT or PE).

Patients randomized to the index group that have a high risk of VTE recurrence after an initial 6 months, but sometimes 3 of 12 months at the treating physician's own discretion, of VKA treatment are advised to continue VKA treatment during the entire follow-up period of 24 months. A priori, we define a high risk of recurrence as > 5% in the first year or > 20% in the first five years (based on the prediction model [22] and experts' consensus [24]). Treating physicians however can overrule this treatment decision if the risk of (major) bleeding during long-term VKA treatment is considered too high. This (usually) will be the case in patients with either one or more of the following characteristics: *history of non reversible bleeding during VKA treatment*, *history of persistently poor anticoagulant control*, *chronic renal* or *hepatic failure*, *history of non cardio-embolic stroke*, patient *older than 75 years* when diagnosed with VTE or if an *informed patient wants to stop VKA treatment*. Referring physicians are allowed to stop VKA treatment in these patients, even if VTE recurrence risk is high. As this is a pragmatic study aimed at improving clinical practice, such patients remain in the study and subsequently enter the follow-up period (after initial treatment of 6 months with VKA conform guidelines).

Patients randomized to the index group that (according to the prediction model) have a low risk of VTE recurrence after stopping VKA treatment (i.e. < 5% in the first year and < 20% in the first five years) are advicsed to stop long-term VKA treatment after a standard initial treatment period of 6 months. After these 6 months, patients are followed-up for 24 months.

Patients randomized to the control group receive care-as-usual, according to current guidelines: usually 6 months of VKA treatment in all patients with a first episode of unprovoked VTE, sometimes longer. The decision whether or not to prescribe long-term VKA treatment is mainly based on physicians' discretion. For comparison reasons, a D-dimer test will be performed after 6 months of initial VKA therapy and one month after discontinuation of VKA. All patients, both controls and index, are followed-up for 24 months.

Recently, a new type of oral anticoagulants (Direct oral anticoagulants (DOACs)) has been registered as therapeutic option in VTE. These DOACs do have the advantage over VKA that no regular INR screening is needed anymore, thus limiting the Thrombosis Service's role. However, the bleeding risk associated with anticoagulant treatment remains and a tailored decision on optimal treatment duration is needed in those patients using DOACs as well. Therefore, we extent the inclusion criteria to those VTE patients switching from VKA to

DOACs during the initial phase of anticoagulant treatment as well. We do not expect any influence on our risk assessment while on anticoagulants: DOACs and VKA both intervene with the formation of fibrin, and a comparable influence on D-dimer levels (suppression while on anticoagulant therapy) is expected.

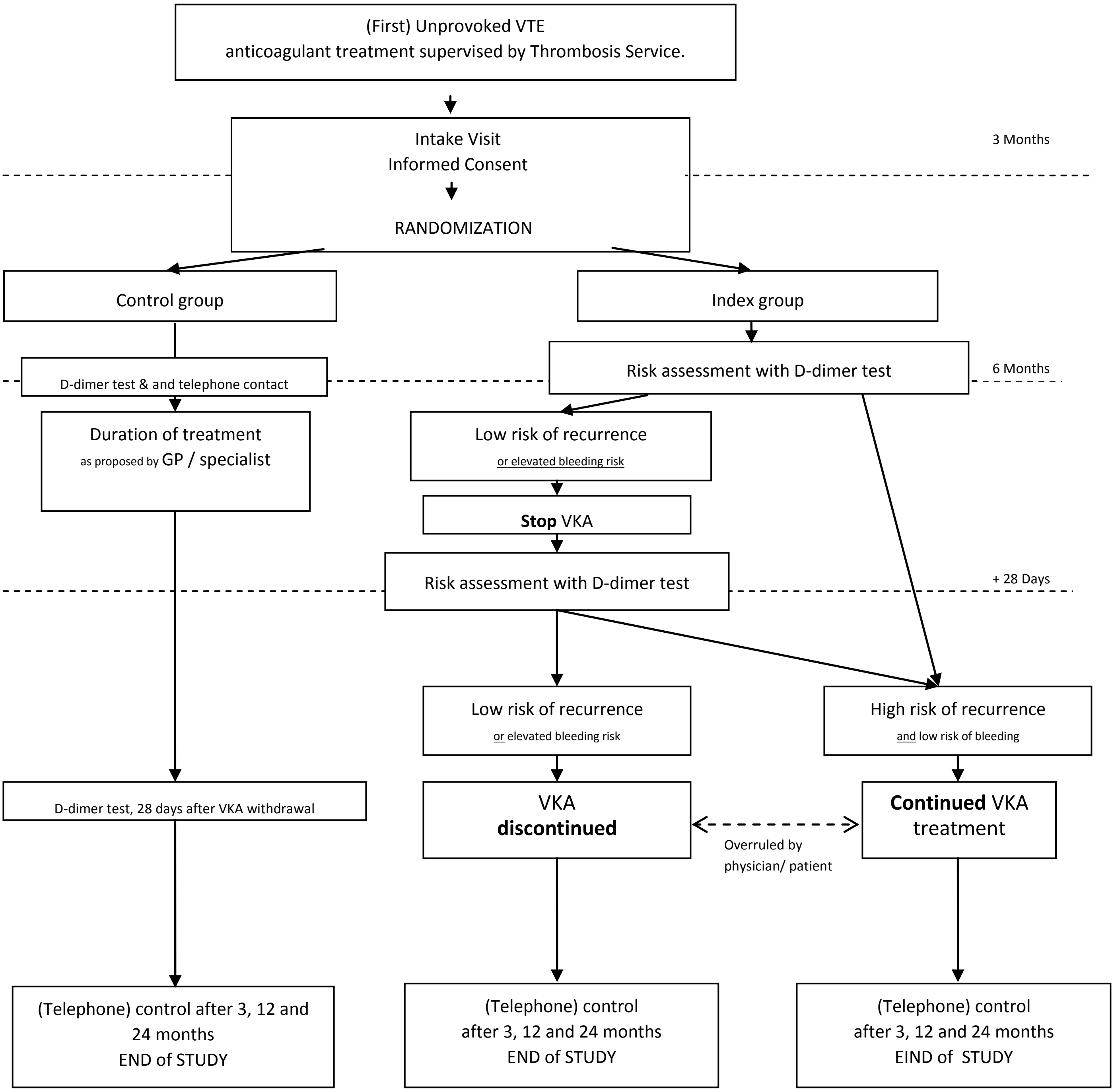

4. STUDY POPULATION

4.1 Population (base):

In The Netherlands, all patients with a confirmed VTE event are referred to a local Thrombosis Service for (long-term) treatment with VKA. In 2008, about 11.000 new DVT patients and 4.000 new PE patients started VKA treatment at these centres. We aim to include patients at 9- 10 thrombosis centres (Amersfoort, Utrecht, Ede, Harderwijk, Zwolle, Rotterdam, Deventer, Enschede, Hilversum and Nijmegen). Each year, these centres start VKA treatment in about 2000 new DVT patients and in 1000 new PE patients. Patients will be recruited at these Thrombosis Services, in close collaboration with the referring physicians and general practitioner.

4.2 Inclusion criteria:

The study population consists of consecutive patients with a venous thromboembolic event (VTE) of the lower extremities or lungs who are referred to the Thrombosis Services for treatment with vitamin K antagonists. All patients have to be 18 years of age or older when inclusion takes place.

4.3 Exclusion criteria:

- Patients with an initial indication for longer treatment with VKA (1 year or longer) as based on the following criteria,
    - recurrent VTE within 10 years
    - atrial fibrillation, prosthetic heart valve, known antiphospholipid antibodies
- Malignancy (latest treatment < 6 months prior to VTE event)
- Patients with provoked VTE, and thus only a short term indication of VKA use (3 months) as defined by presence of:
    - surgery with general or spinal anaesthesia >30 minutes within 3 months prior to diagnosis,
    - lower limb fracture with casting within 3 months prior to diagnosis.
- Pregnancy (or within first 6 weeks after labour),
- Physician states reason not to continue VKA therapy after 6 months of treatment (bleeding risk exceeds risk of recurrence),
- Participation in another trial,
- Not willing or not able to give consent

4.4 Sample size calculation:

Currently, the incidence of recurrent VTE varies in observational studies from 6% to 9% within 12 months.[9,20,21] Only one RCT on a D-dimer guided duration of VKA treatment is available for unprovoked VTE patients[16]. VTE recurrence rates were 15.0% and 2.9% (in about 18 months) in D-dimer positive

patients that were randomized to either discontinuation or prolongation of VKA therapy, respectively. Recalculating these numbers and if care as usual was to be applied to all patients of this RCT, an overall recurrence rate of 9% would be expected [7] with 3.4% if all patients with a positive D-dimer test would have been treated.[12,16] Observational studies show that VTE recurrence rates in D-dimer negative patients is 3.5%. To be on the conservative side, we assume a recurrence rate of 7% in our control group (care-as-usual) and of 3.5% in our intervention group. To determine whether the proposed strategy will lower the recurrence rate from 7% (usual care) to 3.5%, with a power of 80% and alpha of 5% (two sided), at least 692 patients in each arm will be needed. Accounting for loss to follow-up 750 patients in each arm need to be included, this is a total inclusion of 1500 patients.

After an interim analysis in 2014, the sample size was adjusted based on the observed outcomes so far: the recurrence rate in the control group was 9.9% and 4.5% in the intervention group, with an observed risk difference of 5.4%. Assuming that these proportions will not change in the course of the trial, and given the hypothesis that prolonged anticoagulation based on a risk prediction model, we will need patients of 311 per group (alpha 0.05, power 80%, 1-sided testing), and thus a total of 622 patients. Taking into account loss-to-follow-up and alpha spending for this interim analysis, we aim to include a total of 968 patients in our study.

5. TREATMENT OF SUBJECTS

5.1 Investigational treatment:

In patients randomized to the index group, long-term treatment duration will be guided by a prediction model (see flow chart and study design). After 6 (but sometimes 3 or 12) months of initial VKA therapy, a prediction model is applied on the day the patient is instructed to stop VKA treatment (day t=0). If the prediction model demonstrates a low risk of VTE recurrence (< 5% in the first year and < 20% at five years) at both visits (i.e. day t=0 and t=28 days), treatment remains ceased. As soon as the result of the prediction model demonstrates a high risk of VTE recurrence, VKA treatment is advised to be continued or resumed for another 24 months. Referring physicians are allowed to overrule this treatment decision if patients are expected to have a high risk of bleeding during VKA treatment. Possible arguments can be based on one of the following patient characteristics: history of non reversible bleeding during VKA treatment, history of persistently poor anticoagulant control, chronic renal or hepatic failure, history of non cardio-embolic stroke, patient older than 75 years when diagnosed with VTE, an active peptic ulcer or if an informed patient wants to stop VKA treatment.
As the D-dimer result (day 0) can be negative influenced by the VKA treatment [25], we choose to perform a second risk assessment in all ‘ low risk’ patients, 28 days after VKA discontinuation. As soon as one risk assessment yields an increased recurrence risk– including at day 0 (day of withdrawal) – treatment is immediately resumed for the follow-up period of 24 months, to counterbalance the risk of recurrent VTE.

Although a very recent meta-analysis [23] proved that the timing of D-dimer testing after stopping VKA treatment does not affect the ability to distinguish patients with a higher or lower risk for recurrent VTE, we are certainly aware of the risk of recurring VTE during this period when VKA therapy is suspended. The only previous trial on this issue – as we discussed above [16] – performed a D-dimer assay only once at day 28, to determine the (dis)continuation of VKA treatment. Therefore, we will perform the first D-dimer assay at the moment of VKA withdrawal too.

5.2 Escape medication (if applicable): Not applicable.

5.3 Use of co-intervention (if applicable): Not applicable

6. METHODS

6.1 Study parameters/endpoints

6.1.1 Main study parameter/endpoint

The primary outcome is the occurrence (incidence) of recurrent VTE during 24 months of follow-up after the initial 6 months of VKA treatment. Recurrent VTE is defined as proximal DVT and/or fatal or non-fatal PE as confirmed by compression ultrasonography for DVT and by (spiral CT) angiography and/or ventilation-perfusion lung scanning for PE. As (interpretation of the) compression ultrasonography as well as ventilation-perfusion lung scanning and/or (spiral CT) angiography will be part of routine care, interpretation will be independent of (/ blinded to) allocated treatment strategy.

6.1.2 **Secondary study parameters/endpoints:**

Safety and secondary outcomes are the occurrence of major bleedings, quality of life and cost-effectiveness. A major bleeding is defined by *retroperitoneal* or *intracranial* bleeding, a bleeding with *lowering of haemoglobin levels of at least 2.0 g/dl*, or a bleeding for which *transfusion is needed of at least 2 units blood* or *surgical intervention* or *invasive procedures* to stop the bleeding. An independent and blinded adjudication committee will adjudicate each thrombo-embolic recurrent event and death, as well as each potential bleeding event. Minor bleedings (all bleedings not meeting the criteria stated above) will be asked for by filling in the questionnaires well as possible consequences, like non-scheduled contacts with caregivers, "protocol independent" change of VKA therapy, discomfort for the subject or change in quality of life. Examples of minor bleedings are epistaxis > 20 minutes, hematuria, melaena, large hematomas or hematemesis. A cost-effectiveness analysis will be performed from the societal perspective. With a Markov type decision model, costs and health effects as a consequence of difference of treatment are balanced against 'care as usual'. Differences in costs result from different duration of treatment with VKA, from additional D-dimer tests, from treatment of recurrence of VTE and bleeding complications, and from productivity losses associated with recurrent events and bleeding complications. The time horizon used for the economic evaluation will be equal to the study period, i.e. a follow-up period of 24 months after an initial 6 months of VKA treatment. Quality of Life and health care related costs will be assessed at baseline, prior to the intervention and prior to all contact moments during the follow-up period of 24 months, using both generic questionnaires (SF-36; EQ5D, VAS) and a questionnaire on health care consumption& associated costs.

6.2 Randomisation, blinding and treatment allocation:

5 Months after the VTE event, patients will be randomized to either the index group (formal risk assessment including-dimer level to guide VKA treatment) or to the control group (care-as-usual). Blocked, open randomisation will be performed within strata for centre and for type of VTE (DVT or PE).

6.3 Study procedures:

At baseline (preferably 3-6 weeks after VTE event), several patient characteristics will be asked to all patients (including gender, age, type of VTE event, co-morbid diseases and all medication use, and known risk factors for VTE recurrence). One month before the intervention, randomisation will be performed.

After usually 6 months of initial therapy, the intervention phase of the study will start. Patients randomized to the index group may undergo a maximum of 2 risk assessments, for which a maximum of 2 venapunctures for D-dimer testing will be required. For comparison reasons, patients randomized to the control group also undergo 2 venapunctures for D-dimer testing. The first blood sample will be drawn after 6 months of initial treatment (T=0); the second sample will be taken 28 days after discontinuation of VKA treatment. In control patients, this test outcome has no consequences for the treatment duration; the physician and patients remain unaware of the result as the investigators order the test. During the intervention phase of the study as well as at the end of the follow-up phase (at 24 months), all patients will be asked to fill out 'Quality of Life' questionnaires (using the SF36 and EuroQol questionnaires) for the cost-effectiveness analyses. Furthermore the investigators will approach all patients at 3, 12 and 24 months during the follow-up phase of the study. Patients will be asked for the occurrence of recurrent VTE events and possible other changes in health state.

6.4 Withdrawal of individual subjects:

Subjects can leave the study at any time for any reason if they wish to do so without any consequences. The investigator can decide to withdraw a subject from the study for urgent medical reasons.

6.4.1 **Specific criteria for withdrawal (if applicable):** None.

6.5 Premature termination of the study:

The study will be prematurely stopped if the number of recurrences or bleedings in the intervention group during the treatment phase of the study is unacceptable higher than in the controls. For detailed description of reasons to terminate the study, see section 7.4 on the Data Safety Monitoring Board.

7. SAFETY REPORTING

7.1 Section 10 WMO event:

In accordance to section 10, subsection 1, of the WMO, the investigator will inform the subjects and the reviewing accredited METC if anything occurs, on the basis of which it appears that the disadvantages of participation may be significantly greater than was foreseen in the research proposal. The study will be suspended pending further review by the accredited METC, except insofar as suspension would jeopardise the subjects' health. The investigator will take care that all subjects are kept informed.

7.2 Monitoring

This study will be monitored according to the NFU (Dutch Federation of University Medical Centers) risk classification "Minimal excess of negligible risk" by an independent party of the Julius Center Utrecht. Details of this monitoring plan can be found in the appendix "Monitoringsplan VISTA", BKO-2011-053 and the "updated Monitoringsplan VISTA, april 2015".

7.3 Definition of adverse and serious adverse events:

This study investigates the cost-effectiveness of tailoring long-term VKA treatment with a prediction model versus usual care in patients with a (first) VTE event. Although it is expected that this will result in a lower VTE recurrence rate as well as no increase in bleeding complications, patients randomized to this intervention strategy may be at risk of a similar or higher risk of these outcomes. Notably in the time window of (maximally) 28 days when VKA treatment is stopped after an initial treatment of 6 months, patients may be at risk of a VTE recurrence. In addition – based on a prediction model (including positive D-dimer testing) and a subsequent higher anticipated VTE recurrence rate – patients can be treated longer with VKA as compared to usual care. These patients can have a higher risk of bleeding complications. Although rare (1% per patient-year treated), subsequent case fatality of such bleeding complications can be as high as 50%.

Therefore, we define a **Serious Adverse Event** as:
* **fatality** because of a major bleeding event, recurrent Pulmonary Embolism, or any other cause.
* **major bleeding event**: any retroperitoneal or intracranial bleeding event, a bleeding with lowering of haemoglobin of at least 2.0 g/dl (=< 1.24 mmol/L), and/or a bleeding for which transfusion is needed of at least 2 units blood or surgical intervention or invasive procedures to stop the bleeding.
* **Recurrent non-fatal Pulmonary Embolism.**
* Admission to hospital for every other cause (thus, elective or emergency surgery, admission for chest complaints, infection etc.)

An **adverse event** is defined as:
* **Recurrent DVT** (proximal or distal, confirmed by CUS)
* **Minor bleeding**, that is all *clinically relevant* bleedings not meeting the criteria as stated above.
* High bleeding potential, that is konakion prescribed and/ or temporal cessation of VKA therapy

Reporting of SAEs and AEs

Thrombosis services will be asked to report all bleeding complications (sudden VKA withdrawal, Vitamin K prescription) and re-start of VKA therapy (in case of recurrent VTE) immediately to the coordinating investigator. Furthermore, the investigators will ask all patients, referring clinicians and general practitioners to report every possible SAE to the investigators as well.

All *major bleeding* and *fatal* events will be reported through the web portal *ToetsingOnline* to the accredited METC that approved the protocol, within 15 days after the sponsor has first knowledge of the serious adverse event. The DSMB will be informed as well.

SAEs that result in death or are life threatening should be reported expedited. The expedited reporting will occur not later than 7 days after the responsible investigator has first knowledge of the adverse reaction. This is for a preliminary report with another 8 days for completion of the report.

All recurrent PEs and admissions to hospital due to other reasons, will be reported to TO once per 6 months.

All AEs will be filed in the study CRF.

From the moment of intervention (6 months after initial VTE event), SAE's and AE's will count as such. For all details, please find the SAE flow (08-08-2012) in the Appendix.

7.4 Follow-up of adverse events

All adverse events will be followed until they have abated, or until a stable situation has been reached. Depending on the event, follow up may require additional tests or medical procedures as indicated, and/or referral to the general physician or a medical specialist.

7.5 Data Safety Monitoring Board (DSMB):

A DSMB will be installed to assess the progress of the study and in particular the occurrence of (major) bleedings (this is the safety) and the occurrence of VTE recurrences (primary outcome of the study). In order to do so, the DSMB will meet on a regular basis during the study and will have access to all study information (on a patient level). In addition, the DSMB will be notified every 6 months of the number of protocol violations and patient withdrawals (as early indicators of problems related to the study) to assess study integrity and safety. All occurrences of major bleedings and deaths will be notified to DSMB immediately after the event has occurred.

DSMB statistical criteria

During the recruitment and treatment period of the study, interim analyses of main safety events and of any other information on major endpoints that is available will be supplied, in strict confidence, to the DSMB. The DSMB will advise the Trial Steering Committee if, in their view, the randomised comparison in the study has provided "proof beyond reasonable doubt" that one particular treatment strategy is clearly indicated or clearly contraindicated in terms of a net difference in the major outcome measures. Appropriate criteria of proof

beyond reasonable doubt cannot be specified precisely, but a difference of at least three standard deviations (P-value $\approx$ 0.002) in an interim analysis of a major endpoint may be needed to justify halting, or modifying, such a study prematurely. If this criterion were to be adopted, it would have the practical advantage that the exact number of interim analyses would be of little importance, so no fixed schedule is proposed. An interim analysis will take place when 50% of the number of expected bleeding events has been reached. Event rates will be compared between the two treatment arms, taking into account differences in follow-up time between patients at the time of interim analyses. The pre-defined statistical guideline is to consider a difference between the treatment arms with a two-sided p-value of 0.002 as proof beyond reasonable doubt. However, these criteria are used as guidance only. Other safety considerations, such as timing of main adverse events relative to the treatment interruption necessary for the biomarker guided treatment arm, need to be taken into account in the DSMB advice on safety. The advice(s) of the DSMB will be notified upon receipt by the study investigators to the METC that approved the protocol. With this notification a statement will be included indicating whether the advice will be followed.

Members of the VISTA Data Safety Monitoring Board are:
Prof. dr.F.W.A. Verheugt, department of cardiology,OLVG, Amsterdam. Chair of the DSMB
Prof. dr.D.H.Biesma, department of internal medicine St.Antonius Hospital Nieuwegein
Prof. dr. E.W. Steyerberg, clinical epidemiologist, Erasmus MC Rotterdam

8. STATISTICAL ANALYSIS

8.1 Descriptive statistics:

The aim of the main analysis is to compare the incidence of the primary outcome (VTE recurrence incidence within 24 months after discontinuation of anticoagulant therapy) in both patient groups. Baseline differences between the two randomised groups are determined. The primary outcome will be presented as relative risk with 95% confidence interval. Also the risk difference with 95% confidence interval and the number needed to treat will be estimated. The analytical steps will also be performed for the bleeding outcome.

8.2 Multivariable analysis:

Kaplan Meier (and Cox survival) analysis will be used to analyze the time to a recurrent VTE event. The analysis will be stratified (adjusted) for centre, and type of VTE (DVT or PE). Point and interval estimates and p-values for the primary and the safety outcomes will be adjusted for the repeated testing by interim analyses. All analyses will be done according to the intention to treat principle.

For the cost-effectiveness analyses first order Monte Carlo analysis will be used. Cycle length will be one month. One way sensitivity analysis will be performed to establish the separate effect of model parameters on the results of the analysis. The model parameters will be varied over a plausible range. The influence of parameter uncertainty will be further tested using probabilistic sensitivity analysis.

8.3 Interim analysis (if applicable)

Most recurrences will happen in the two year follow up phase. For deaths and bleeding events, the DSMB will monitor the study on regular base. For details, see section on DSMB in section 7.4.

9. ETHICAL CONSIDERATIONS

9.1 Regulation statement:

The study will be conducted according to the principles of the Declaration of Helsinki (latest version as adopted by the 59th WMA assembly, Seoul October 2008) and in accordance with the Medical Research Involving Human Subjects Act (WMO).

9.2 Recruitment and consent:

In the Netherlands, each patient with a confirmed VTE event is referred to a Thrombosis Service by the referring physician (specialist or general practitioner (GP)). Two routes will be used to recruit participants:

- During the (regular) intake of the patient at the Thrombosis Service, a nurse from the Thrombosis Service asks if the patient has any objections to receive a phone call by a member of the investigators team (research nurse) to receive information on the study described above. Subsequently, all patients that provide their telephone number are then called by the research nurse to provide information on the study and – if no contra-indications are present – the nurse will plan a VISTA intake visit, at home or (preferably) at the Thrombosis Service or general practice.
- After the regular intake, a nurse of the Thrombosis Service will contact all referred patients with the indication VTE to be invited to participate: he/ she provides information on the study and – if no contra-indications are present – the nurse will plan a VISTA intake visit, at home or (preferably) at the Thrombosis Service or general practice.

During this intake visit (during the initial VKA treatment phase of 6 months), informed consent is asked as well some additional information on patient and VTE characteristics (baseline). In addition, the research nurse informs the referring physician (both specialist and GP) that his or her patient is candidate to be included in the VISTA study and will ask consent and some additional information on patient- or VTE characteristics. Patients are randomized to either 'care-as-usual' or prediction model guided treatment 1 month prior to the intervention.

9.3 Compensation for injury:

The sponsor has a liability insurance which is in accordance with article 7, subsection 6 of the WMO.

The sponsor (also) has an insurance which is in accordance with the legal requirements in the Netherlands (Article 7 WMO and the Measure regarding Compulsory Insurance for Clinical Research in Humans of 23th June 2003). This insurance provides cover for damage to research subjects through injury or death caused by the study.

1. € 450.000,-- (i.e. four hundred and fifty thousand Euro) for death or injury for each subject who participates in the Research;
2. € 3.500.000,-- (i.e. three million five hundred thousand Euro) for death or injury for all subjects who participate in the Research;

3. € 5.000.000,-- (i.e. five million Euro) for the total damage incurred by the organisation for all damage disclosed by scientific research for the Sponsor as 'verrichter' in the meaning of said Act in each year of insurance coverage.

The insurance applies to the damage that becomes apparent during the study or within 4 years after the end of the study.

9.4 Outlining the responsibilities during the study

The initial 6 months of VKA treatment is the treatment as suggested by the physician who referred an individual patient to the Thrombosis Service with the indication VTE. Since no intervention will take place during this period, the responsibility for the treatment is considered to be the same as usual: according to the Dutch Health Act WGBO the referrer is the treating physician. VISTA will not intervene in the treatment strategy of the control group after randomization. The referrer proposes the optimal duration of therapy, after discontinuation, the GP will be in charge of the further (preventive) management. The duration of VKA therapy in the index group can deviate from the initial management plan as stated by the physician who referred the patient to the Thrombosis Service. The investigators will notify each physician if this occurs. Without explicit objection of this physician, eventual prolonged duration of VKA therapy will be coordinated by the medical director (or leader) of the Thrombosis Service, in close collaboration with the investigators. After 24 months of VKA therapy, in consultation with the GP and specialist, even longer duration of therapy will assessed, taking into account the 2 year period of treatment, possible new bleeding risk factors, etc. When treatment is stopped, the GP is in charge for future management.

10. ADMINISTRATIVE ASPECTS AND PUBLICATION

10.1 Handling and storage of data and documents:

All data will be stored by the data management department of the Julius Center for Health Sciences and Primary Care (UMC Utrecht) and kept for 15 years. Data will be stored encoded as data regarding patient identification will be replaced by a patient study ID number, if possible during the study period. Due to safety reasons, during the intervention phase of the study, it will not be possible to completely replace all patient identification data since the investigators need access to patient details in case of contact with GPs or other health care workers. Data will be analysed by the investigators of the study.

10.2 Amendments:

Amendments are changes made to the research after a favourable opinion by the accredited METC has been given. All amendments will be notified to the METC that gave a favourable opinion.

10.3 Annual progress report:

The investigator will submit a summary of the progress of the trial to the accredited METC once a year. Information will be provided on the date of inclusion of the first subject, numbers of subjects included and numbers of subjects that have completed the trial, serious adverse events/ serious adverse reactions, other problems, and amendments.

10.4 End of study report:

The investigator will notify the accredited METC of the end of the study within a period of 8 weeks. The end of the study is defined as the last patient's last visit.

In case the study is ended prematurely, the investigator will notify the accredited METC, including the reasons for the premature termination.

Within one year after the end of the study, the investigator will submit a final study report with the results of the study, including any publications/abstracts of the study, to the accredited METC.

10.5 Public disclosure and publication policy:

The researchers will seek publication of their results in international peer reviewed journals. There have been no restrictions placed upon publication by the sponsors (ZON-MW- Doelmatigheid 171002214, Bayer Shering Pharma) of this study.

11. REFERENCES

[1] Hull R, Delmore T, Genton E, Hirsh J, Gent M, Sackett D et al. Warfarin sodium versus low-dose heparin in the long-term treatment of venous thrombosis. N Engl J Med 1979; 301(16):855-858. 5

[2] Prandoni P, Lensing AWA, Cogo A, Cuppini S, Villalta S, Carta M et al. The long-term clinical course of acute deep venous thrombosis. Ann Intern Med 1996; 125:1-7.

[3] Bounameaux H, Perrier A. Diagnostic approaches to suspected deep vein thrombosis and pulmonary embolism Hematol J. 2003;4(2):97-103.

[4] Dalen JE, Alpert JS. Natural history of pulmonary embolism. Prog Cardiovasc Dis 1975;17:257-70

[5] Diagnosis, Prevention and Treatment of VTE and Secondary Prevention of Arterial Thrombosis (Richtlijn Diagnostiek, Preventie en Behandeling van Veneuze Tromboembolie en Secundaire Preventie Arteriële Trombose). 2009 Kwaliteitsinstituut voor Gezondheidszorg CBO, Utrecht, The Netherlands. www.cbo.nl

[6] Douketis JD, Kearon C, Bates S, Duku EK, Ginsberg JS. Risk of fatal pulmonary embolism in patients with treated venous thromboembolism. JAMA 1998;279(6):458-62.

[7] van Dongen CJ, Vink R, Hutten BA, Buller HR, Prins MH. The incidence of recurrent venous thromboembolism after treatment with vitamin K antagonists in relation to time since first event: a meta-analysis. Arch Intern Med 2003;163(11):1285-93.

[8] Cochrane Database Syst Rev 2006;(1):CD001367.

[9] Kearon C, Kahn SR, Agnelli G, Goldhaber S, Raskob GE, and Comerota AJ. Antithrombotic Therapy for Venous Thromboembolic Disease. American College of Chest Physicians Evidence-Based Clinical Practice Guidelines (8th Edition) Chest 2008;133;454S-545S

[10] Hyers TM. Duration of Anticoagulation in Venous Thromboembolism. Arch Intern Med. 2003; 163: 1265-6.

[11] Agnelli G, Becattini C. Treatment of DVT: how long is enough and how do you predict recurrence. J.Thromb Thrombolysis. 2008; 25:37-44.

[12] Palareti G, Legnani C, Cosmi B, Guaazzaloca G, Pancani C, Coccheri S. Risk of Venous Thromboembolism recurrence: High Negative Predictive Value of D-dimer Performed after Oral Anticoagulation Is Stopped. Throm Haemost 2002;87:7-12.

[13] Kuruvilla J, Wells PS, Morrow B, MacKinnon K, Keeney M, Kovacs MJ. Prospective assessment of the natural history of positive D-dimer results in persons with acute venous thromboembolism (DVT or PE). Thromb Haemost 2003;89:284-7.

[14] Palareti G, Legnani C, Cosmi B, Valdre ´ L, Lunghi B, Bernardi F, et al. Predictive value of D-dimer test for recurrent venous thromboembolism after anticoagulation withdrawal in subjects with a previous idiopathic event and in carriers of congenital thrombophilia. Circulation. 2003;108:313-8.

[15] Eichinger S, Minar E, Bialonczyk C, Hirschl M, Quehenberger P, Schneider B et al. D-dimer levels and risk of recurrent venous thromboembolism. JAMA 2003;290(8):1071-4.

[16] Palareti G, Cosmi B, Legnani C, Tosetto A, Brusi C, Ioro A, Pengo V, Ghirarduzzi A et al. D-dimer testing to determine the duration of anticoagulation therapy. N Eng J Med 2006;355:1780-9.

[17] Shrivastava S, Ridker PM, Glynn RJ, Goldhaber SZ, Moll S, Bounameaux H, et al. D-dimer, factor VIII coagulant activity, low-intensity warfarin and the risk of recurrent venous thromboembolism. J Thromb Haemost. 2006;4:1208-14.
[18] Tait R, Lowe GDO, McColl MD, McMahon AD, Robertson L, King L, et al. Predicting risk of recurrent venous thrombosis using a 5-point scoring system including ﬁbrin D-dimer. J Thromb Haemost. 2007;5(Suppl 2):60.
[19] Poli D, Antonucci E, Ciuti G, Abbate R, Prisco D. Combination of D-dimer, F1 2 and residual vein obstruction as predictors of VTE recurrence in patients with ﬁrst VTE episode after OAT withdrawal [Letter]. J Thromb Haemost. 2008;6:708-10.
[20] Baglin T, Palmer CR, Luddington R, Baglin C. Unprovoked recurrent venous thrombosis: prediction by D-dimer and clinical risk factors. J Thromb Haemost. 2008;6:577-82.
[21] Verhovsek M, Douketis JD, Yi Q, Shrivastava S, Tait RC, Baglin T, Poli D, Lim W. Systematic Review: D-Dimer to Predict Recurrent Disease after Stopping Anticoagulant Therapy for Unprovoked Venous Thromboembolism. Ann Int Med 2008;149(7):481-90.
[22] Eichinger S, Heinze G, Jandeck LM, Kyrle PA. Risk Assessment of Recurrence in Patients with Unprovoked Deep Vein Thrombosis or Pulmonary Embolism. The Vienna Prediction Model. Circulation 2010;121:1630-1636.
[23] Douketis J, Tosetto A, Marcucci M, Baglin T, Cushman M, Eichinger S, Palareti G, Poli D, Campbell Tait R, Ioro A. Patient-Level Meta-analysis: Effect of Measurement Timing, Threshold, and Patient Age on Ability of D-dimer Testing to Assess Recurrence Risk After Unprovoked Venous Thromboembolism. Ann Int Med 2010;153:523-531.
[24] Kearon C, Iorio A, Paraleti G. and On behalf of the subcommittee on control of anticoagulation of the SSC of the ISTH, Risk of recurrent venous thromboembolism after stopping treatment in cohort studies: recommendation for acceptable rates and standardized reporting. Journal of Thrombosis and Haemostasis 2010, 8: 2313–2315.
[25] Ombandza-Moussa E, Samama MM, Horellou MH, Elalamy I, Conard J. Potential use of D-dimer measurement in patients treated with oral anticoagulant for a venous thromboembolic episode. Int Angiol. 2003 Dec;22(4):364-9.
