# Supplementary material for: Effect of tailoring anticoagulant treatment duration by applying a recurrence risk prediction model in patients with venous thromboembolism compared to usual care: A randomized controlled trial
Source: PLoS Med. 2020 Jun 26;17(6):e1003142. doi: 10.1371/journal.pmed.1003142 (PMC7319277; doi:10.1371/journal.pmed.1003142)
Supplement: S2 Text — (DOCX) [file pmed.1003142.s006.docx]

**S2 Text: Additional analyses prompted by PLOS Medicine reviewers:**

**Questions related to protocol non-adherence:**

**R1: When restricting the analysis to patients with a high estimated probability of adherence, please report the degree of comparability between the index and control group. i.e. were they well matched or did some baseline imbalance remain between the two groups after selecting those with a high probability of adherence? Please consider adding the corresponding baseline table to the online supplement. In case of baseline imbalances (e.g. standardised difference greater than 0.1), please consider adjusting the analysis.**

**R2: The secondary analysis, which was not pre-planned, is of interest as these estimates may reflect a more realistic effect of what would be seen in everyday clinical practice. However, the implementation of the approach is lacking details. The authors are encouraged to consult this reference DOI: 10.1056/NEJMsm1605385, which outlines an appropriate analytic plan to conduct per-protocol analyses specifically for pragmatic trials that minimizes the risk of selection bias in relation to censoring. Additionally, the following sentence is unclear: (p7) "we used patients within the 75th quartile of predicted probabilities of protocol adherence." Was the data restricted based on the propensity score? If so, it would be important to characterize the patients who did not enter this analysis - what were the main differences in terms of baseline characteristics? Perhaps an approach like inverse probability of treatment (or rather protocol adherence) weighting could allow for a more
efficient use of the available data?**

*Answer: We thank the reviewer for these comments. These are very constructive remarks, indeed further improving our findings and in particular helps in understanding the results of our trial. First, prompted by this remark, we below present the standardised mean differences over the 10 imputed datasets in our analysis including patients with a probability of being adherent to our the VPM recommendations (75^th^ percentile, see main text). As can be appreciated, standardised mean differences for sex, type of VTE (PE versus DVT), and oral contraceptive use are lower than 0.1 for all imputed datasets. However, for D-dimer this indeed does not seem to be the case, indicating imbalance for D-dimer values.*

*As suggested by the reviewer, we therefore adjusted our analyse. This in fact was also suggested by reviewer #2 (question 3), suggesting to perform inverse probability of treatment weighting (IPW) analysis. Prompted by this remark, as well as the observed imbalance (for D-dimer only) in our current analysis to adjust for protocol non-adherence, we additionally did this IPW analysis. This analysis yielded similar results, with a RR of 1.0 (95% CI 0.95-1.05, p=0.93).*

*Given that both analyses yield similar results (and in fact are on the line of unity), we therefore believe that our main inference of not finding an effect of applying the VPM in our study domain is unlikely to be fully explained due to non-adherence to our protocol. This issue is now discussed in our discussion section of our manuscript, reading as follows (changed, using track changes).*

**Questions related to VPM validation in patients treated with anticoagulants:**

**R1: I am confused by the fact that the predictive performance analysis excluded patients who continued anticoagulant treatment. In patients randomised to the index group, only those at higher risk (according to the Vienna prediction model itself) would have continued anticoagulant treatment. Therefore, excluding these patients limits the validation exercise to only those at lower risk. Please confirm/clarify.**

**R2: What is the argument for assessing performance only among patients who did not receive prolonged treatment? This would be a selected group of patients with a presumed low recurrence risk. Calibration in high risk patients would be of clinical relevance.**

*Answer: This is an excellent question. Indeed, we now analysed the predictive performance (discrimination and calibration) only in patients that did not prolong anticoagulant treatment, i.e. in 377 patients from the control arm of our trial and 317 patients from the index arm (total 694 patients). This was done deliberately as prolonged anticoagulant treatment (obviously) lowers the risk of recurrent VTE, thereby leading to an apparent (and biased) overestimation of the predicted risk by the Vienna prediction model (VPM). Nevertheless, we also agree that excluding patients that continue anticoagulation altogether may lead to a scenario where we only validate the VPM in a lower risk population.*

*Therefore, prompted by this remark, we now added several additional analyses. First, we repeated all analyses on the predictive performance of VPM in all patients, thus also including those having received anticoagulant treatment. Results of calibration plots are shown below, showing similar results as our initial analysis including only patients not receiving anticoagulant treatment. Discrimination results of including only non-treated patients was 0.76 (95% CI 0.69-0.83) versus 0.69 (95% CI 0.62-0.77) when using all available patient information (including treated patients).*

*However, clearly, the VPM still underestimates risk in patients with a high predicted risk. We believe this further strengthens our observation that VPM likely underestimates risk in high risk groups. High risk patients are more likely to receive treatment and thus including these patients in our analyses would lower the observed risk in this risk category potentially leading to an underestimation of risk, whereas we still observe an overestimated risk.*

*Next, we performed a more advanced approach to better account for any bias induced by excluding patients on prolonged anticoagulant treatment. Hereto, we used the relatively recently developed methods as proposed by Pajouheshnia and co-workers (see BMC medical research methodology; 2017 Dec;17(1):103). This method uses inverse probability weighting (IPW). We first fitted a propensity score model (psm) regressing the likelihood of receiving prolonged anticoagulant treatment (dependent variable) using the predicted risk of recurrent VTE from the VPM as independent variable. Next, all analyses in the non-treated population are repeated, yet each non-treated patient receives a weight, based upon the inverse of the propensity of being treated, in fact: 1/(1-psm). As such, non-treated patients with a higher likelihood (propensity) of prolonging anticoagulant treatment (thus more likely the higher risk patients) receive more weight in these analyses, thereby accounting for the rightfully mentioned observation from the reviewer of only using low-risk patients in our validation analyses. The inferences of these analyses are also similar to our initial analyses, still showing underestimated risks notably for those with higher predicted risks; see below.*

*In terms of discrimination, this analysis indeed showed somewhat better performance of the VPM (c-statistic 0.80, 95% CI 0.75-0.84), yet this would in our view not strongly influence our inferences.*

**Questions related to antithrombotic treatment in DOAC-era:**

**R3: This study in fact investigates 2 things at the same time: the performance of the VPM and the related antithrombotic strategy. The authors now conclude that the absence of a difference in risk is related to the application of the model. However, it could also simply be due to insufficient treatment related to the result of the model. Theoretically the recurrence risk in both study arms should be equal. So, to find a difference in actual VTE occurrence, a strategy should have been in place that would strongly impact this risk. This has not really happened, so it seems, considering that the rate of prolonged treatment was in fact low in both arms, although a bit higher in the intervention arm: about 25 vs 10% (if I infer correctly from fig 2). So all effect from applying the model should have come from this small difference in treated subjects, which seems implausible. This option, of an insufficient treatment difference between the arms, could receive more attention in the discussion.**

**In fact I think it could be modelled (e.g. as in a Mendelian Randomisation analysis) how many more cases could have been prevented if the difference should have been larger between the groups (and likewise the bleedings). This would clearly not be the same as in a true RCT but it might shed some light on the respective role of the VPM and the applied treatment strategy in the observed results of this study.**

*Answer: We would like to thank the reviewer for these questions as they are highly relevant. They strongly relate to each other, therefore, we will answer them together. Indeed, as also mentioned by reviewer 2, the current clinical practice in the DOAC-era is that patients with unprovoked VTE are almost always offered the option (or even recommendation) to prolong anticoagulation to minimize their risk of recurrent VTE. Paradoxically, this recommendation is even stronger because validated tools to predict the risk of recurrence are not available, notwithstanding the fact that we also know that recurrence risk is mixed in the population of patients with unprovoked VTE. So, in terms of clinical implications, we believe it indeed would be informative to explore what could have happened in our trial in the current antithrombotic landscape of prolonging (DOAC) treatment in most patients.*

*Hereto, as suggested by the reviewer, we tried to model several scenario’s in a hypothetical cohort of 1000 patients with unprovoked VTE, followed-up for 5 years. First, based upon our own observations, we know that the proportion of patients categorized in the following risk strata of annualized VTE recurrence risk of 0-2%, 2-4%, 4-5%, or >5% are 4.9%, 35.1%, 17.1%, and 42.9%, thus 49, 351, 171, and 429 patients in a hypothetical cohort of 1000 patients, respectively. Based upon our VISTA data, we also know the observed annualized rates of recurrent VTE in these risk strata if anticoagulant treatment is withheld, i.e. 0.0%, 2.5%, 6.7%, and 14.4%, respectively. We know from the NOAC extension trials that these risks of recurrent VTE can be lowered with an observed hazard ratio in these trials of (about) 0.25, thus yielding annualized rates of recurrent VTE if managed with prolonged NOAC-treatment to be 0.0%, 0.63%, 1.68%, and 3.6%, respectively. Now, let’s assume the annualized rates of recurrent VTE to be constant over a 5-year period. This is a conservative assumption, as we know that the rates of recurrent VTE are highest in the first year after the initial event and typically a little bit lower in the subsequent years. Using these data, we can estimate the number of VTE events over a 5 year period in our hypothetical cohort of 1000 VTE patients for the following scenario: (i) prolong treatment in all patients, (ii) prolong only if the estimated risk of recurrence is >2%, (iii) prolong only if the estimated risk of recurrence is >4%, (iv) prolong only if the estimated risk of recurrence is >5%, or (v) do not prolong anticoagulant treatment altogether. In fact, the number of recurrent VTE events for these scenario’s would be 103, 103, 135, 178, and 410 events; or compared to prolonging treatment in all patients (first scenario): 0, 0, 33, 76, and 308 additional events, respectively.*

*Similarly, we can estimate the number of major bleeding events induced in these different scenario’s. Unfortunately, the annualized rates of major bleeding in VTE patients on prolonged DOAC-treatment is less certain, so let’s assume the number of major bleeding events for three different annualized rates of major bleeding: 0.5%/year, 1.0%/year, and 1.5%/year. Over a 5-year period, we can then calculate the number of major bleeding events for these different rates of major bleeding. All these numbers are depicted in the following table, that is now also included as a Supplementary Table S3.*

**Supplementary Table S3: Clinical outcomes in a hypothetical cohort of 1000 VTE patients, followed-up for 5 years**

| **Treatment strategy** | **Number of treated patients** | **Recurrent VTE** |  | **Major bleeding** |  |
| --- | --- | --- | --- | --- | --- |
|  |  | *number* | *additional VTE* |  | *number* |
| **Treat all patients** | 1000 | 103 | 0 | Rate 0.5% per year | 25 |
|  |  |  |  | Rate 1.0% per year | 50 |
|  |  |  |  | Rate 1.5% per year | 75 |
| **Treat only >2% risk/year** | 950 | 103 | 0 | Rate 0.5% per year | 24 |
|  |  |  |  | Rate 1.0% per year | 47 |
|  |  |  |  | Rate 1.5% per year | 71 |
| **Treat only >4% risk/year** | 600 | 135 | 33 | Rate 0.5% per year | 13 |
|  |  |  |  | Rate 1.0% per year | 27 |
|  |  |  |  | Rate 1.5% per year | 40 |
| **Treat only >5% risk/year** | 430 | 178 | 76 | Rate 0.5% per year | 9 |
|  |  |  |  | Rate 1.0% per year | 17 |
|  |  |  |  | Rate 1.5% per year | 26 |
| **Treat no patients** | 0 | 410 | 308 | Rate 0.5% per year | 0 |
|  |  |  |  | Rate 1.0% per year | 0 |
|  |  |  |  | Rate 1.5% per year | 0 |

*Subsequently, we can plot these numbers graphically with on the x-axis the threshold above which prolonged anticoagulant treatment is prescribed, and on the y-axis the number of additional VTE events and major bleeding events (for three different rates of major bleeding) induced by these different strategies. This graphical plot would then be as follows:*

*This graphical plot illustrates that indeed somewhere between the threshold of 3% and 4% the number of additional VTE events induced by treating less patients with unprovoked VTE equals the number of major bleeding events induced due to prolonged NOAC-treatment.*

*We now added this Figure to the main body of manuscript, and updated our section on clinical implications describing these issues, using track changes.*
