# Supplementary material for: Effect of tailoring anticoagulant treatment duration by applying a recurrence risk prediction model in patients with venous thromboembolism compared to usual care: A randomized controlled trial
Source: PLoS Med. 2020 Jun 26;17(6):e1003142. doi: 10.1371/journal.pmed.1003142 (PMC7319277; doi:10.1371/journal.pmed.1003142)
Supplement: Attachment — Submitted filename: Rebuttal_R1.docx [file pmed.1003142.s007.docx]

**Requests from the editors:**

**1. Please ensure you provide article meta-data such as financial information, competing interests and data availability statements.**

*Answer: This has now been altered accordingly and we believe we now included all relevant meta-data, using track changes.*

**2. Please revise your title according to PLOS Medicine's style. Your title must be nondeclarative and not a question. It should begin with main concept if possible. "Effect of" should be used only if causality can be inferred, i.e., for an RCT. Please place the study design ("A randomized controlled trial," "A retrospective study," "A modelling study," etc.) in the subtitle (i.e., after a colon).**

*Answer: This has now been altered; the full title now is: “Effect of tailoring anticoagulant treatment duration by applying a recurrence risk prediction model in patients with venous thrombo-embolism compared to usual care: a randomized controlled trial.”*

**3. Please structure your abstract using the PLOS Medicine headings (Background, Methods and Findings, Conclusions). Could you also include a sentence to describe unprovoked venous thrombo-embolism.**

*Answer: This is now conform PLOS Medicine headings, and a definition of unprovoked was added, i.e. without the presence of apparent transient risk factors like recent surgery.*

**4. Please complete the CONSORT checklist and ensure that all components of CONSORT are present in the manuscript, including how randomization was performed, allocation concealment, blinding of intervention, definition of lost to follow-up, and power statement. The CONSORT checklist should be cited within the methods section and page numbers should be removed as these are likely to change during publication. Please instead refer to paragraphs and sections.**

*Answers: This has now been added accordingly.* 

**5. Abstract Methods and Findings:**

Please ensure that all numbers presented in the abstract are present and identical to numbers presented in the main manuscript text.

*Answer: Numbers in the abstract are identical to those mentioned in the main manuscript text.*

Please include the study design, population and setting, number of participants, years during which the study took place, length of follow up, and main outcome measures.

*Answer: This has been altered accordingly, using track changes.*

Please quantify the main results (with 95% CIs and p values)

*Answer: These have now been added accordingly, using track changes.*

Please include a summary of adverse events if these were assessed in the study.

*Answer: The main adverse events in this trial is the occurrence of bleeding events. These are included in the abstract.*

In the last sentence of the Abstract Methods and Findings section, please describe the main limitation(s) of the study's methodology.

*Answer: This has been altered accordingly, using track changes.*

**6. Abstract primary outcome does not match the primary outcome noted in the clinical trial registry, please clarify.**

*Answer: We apologize for the unclarity here. Indeed, it appears that ‘major bleeding during 24 months’ is by mistake (typo) explained as primary and secondary outcome on the summary section on the website (www.trialregister.nl), whereas later on the same website it is correctly explained that ‘recurrent VTE during 24 months’ is the primary outcome of our RCT and major bleeding is the secondary outcome. This is accordingly also explained in the methods section of our manuscript and mentioned in the (now attached) full original protocol of our trial.*

**7. Please provide brief information about the VISTA study and introduce the term on first view.**

*Answer: We are afraid VISTA is not an acronym, but rather the name given for this study without any reference to additional meaning. The name itself, as well as the need to perform this study, is now first introduced in the introduction part of our manuscript.*  

**8. Line 102, study protocol URL appears to be the journal URL, please provide the study protocol as SI files or provide the correct link.**

*Answer: Thanks for pointing this out. We attached the original and full protocol as PDF in Editorial Manager, to be added as an online appendix to our paper that – when accepted – can be made publicly available via the online website of PLOS Medicine. This has now been clarified in the manuscript.*

**9. The information about ethics approval and written informed consent should ideally be provided together within the methods section.**

*Answer: This has now been altered accordingly.*

**10. Please provide dates of patient recruitment and follow up more clearly in the methods section.** 

*Answer: This has now been replaced from the beginning of the results section to the methods section, using track changes.*

**Comments from the reviewers:**

***Reviewer #1 (Laurent Billot):***

This article presents the results of a randomised trial testing whether using the Vienna prediction model decreases the incidence of recurrent venous thrombo-embolism (VTE) compared to usual care in patients with previous unprovoked VTE. It also seeks to assess the predictive ability of the Vienna model by treating the study as a cohort. While the results are clearly presented and I have no major concern, I have a number of minor comments listed below:

*Answer: We thank the reviewer for his very positive remarks.*

Minor comments:

**1. Please update the title to include the study design as per PLOS Medicine conventions.**

*Answer: This was also asked by the editors and has been changed accordingly.*

**2. In the conclusion of the abstract, please consider changing the last sentence to make the statement less definitive/certain. For example, by writing: "in those with a low predicted risk of recurrence, the observed rate was also low; suggesting that it might be safe to stop anticoagulant treatment in these patients".**

*Answer: This is a very good suggestion, we changed the final sentence of the abstract according to the reviewers’ suggestion.*

**3. Please clarify whether the primary outcome (see lines 167-168) was measured 24 months after randomisation or 24 months after the end of this initial treatment phase. I believe this is in fact the same time point but it would be good to make it more explicit when defining the outcome.**

*Answer: This is a very good question. Indeed, randomization took place at the end of the initial treatment phase. This was deliberately done as in a very small subgroup of patients with unprovoked VTE additional clinical information becomes available during the first months of treatment that will influence any decision on prolonged treatment. Examples include e.g. finding an occult malignancy during the initial treatment phase (always indication for prolonged treatment) or the patients’ wish to not prolong anticoagulant treatment due to fear of bleeding or side-effects. Thus, in these patients randomization to our risk-tailored approach would not have been feasible or ethical as in fact in these patients there is no clinical equipoise/dilemma whether or not to prolong anticoagulant treatment. This in fact happened in 74 patients, with reasons explained in the flowchart (Figure 1).*

*So, yes, the primary outcome was measured 24 months after randomization, which in fact indeed is the same as 24 months after the end of the initial treatment phase. This has now been explained in more detail in the section on ‘randomization and blinding’ as well as in the section on ‘follow-up appointments’ using track changes.*

**4. Please clarify whether outcome assessment was performed and/or adjudicated in a blinded manner and, if not, please discuss the potential risks associated with reporting/ascertainment bias.**

*Answer: In this pragmatic trial, outcomes were adjudicated in a non-blinded manner. Rather, all available hospital discharge information was routinely scrutinized to classify outcome events. This is now clarified in the section on outcome measures, as follows (using track changes): ‘Outcome assessment was done after scrutinizing all available hospital discharge information and recurrent VTE was defined as such if a patient was managed (with anticoagulation) accordingly. Thus, given the pragmatic nature of our trial, outcomes were not adjudicated blinded to randomized allocation.’*

*We agree with the reviewer that non-blinded outcome adjudication may be associated with reporting bias, this is potential outcomes in the index arm of the trial may less likely be attributed as such, compared to potential events occurring in the control arm, due to an anticipated belief that the intervention must be successful. We believe the potential influence of this bias is likely very low, or even negligible, in our trial: hospital specialists were not actively involved in this trial as participating study sites, nor was there (thus) any motive for them to favor the outcomes in the intervention or control arm of our trial. Nevertheless, we agree this should be discussed in the limitations section of our manuscript, where we now added a paragraph explaining the potential bias associated with non-blinded outcome adjudication, using track changes.*

**5. Line 221 mentions imputed datasets; however, data imputation is not discussed prior to this statement (it is mentioned in the last sentence of the methods). Please clarify the nature of the imputation including variables imputed, variables included in the imputation model and the number of imputed datasets. Please also indicate whether the imputation was pre-specified in the protocol or analysis plan or post-hoc.**

*Answer: We apologize for the unclarity here. Imputation was performed for two purposes, namely (i) to fit a propensity model to explore the impact of non-adherence to the recommended VPM strategy, and (ii) to validate the VPM to predict VTE recurrence. In both cases, D-dimer values were imputed for 251 patients (28.4%), notably missing in patients from the control arm of our trial. We now added a new short paragraph on missing values, just below the paragraph on statistical analyses and power calculations, as follows:*

*‘Both for the explorative analysis on the impact of non-adherence to the recommended strategy based upon the Vienna prediction model (fitting of the propensity model, see above) as well as the validation analysis of the Vienna prediction model, missing values for D-dimer were imputed using chained equations. D-dimer levels were missing in 251 patients (28.4%), and these missing values were imputed using all available information including the patients’ baseline characteristics as well as the observed outcome. Ten imputed sets were created and the results of analyses were pooled based on methodological recommendations.’*

*Imputation was not described as pre-specified in the protocol of our paper but deemed necessary based upon the observed number of missing D-dimer values.*

**6. When restricting the analysis to patients with a high estimated probability of adherence, please report the degree of comparability between the index and control group. i.e. were they well matched or did some baseline imbalance remain between the two groups after selecting those with a high probability of adherence? Please consider adding the corresponding baseline table to the online supplement. In case of baseline imbalances (e.g. standardised difference greater than 0.1), please consider adjusting the analysis.**

*Answer: We thank the reviewer for these comments. These are very constructive remarks, indeed further improving our findings and in particular helps in understanding the results of our trial. First, prompted by this remark, we below present the standardised mean differences over the 10 imputed datasets in our analysis including patients with a probability of being adherent to our the VPM recommendations (75^th^ percentile, see main text). As can be appreciated, standardised mean differences for sex, type of VTE (PE versus DVT), and oral contraceptive use are lower than 0.1 for all imputed datasets. However, for D-dimer this indeed does not seem to be the case, indicating imbalance for D-dimer values.*

*As suggested by the reviewer, we therefore adjusted our analyse. This in fact was also suggested by reviewer #2 (question 3), suggesting to perform inverse probability of treatment weighting (IPW) analysis. Prompted by this remark, as well as the observed imbalance (for D-dimer only) in our current analysis to adjust for protocol non-adherence, we additionally did this IPW analysis. This analysis yielded similar results, with a RR of 1.0 (95% CI 0.95-1.05, p=0.93).*

*Given that both analyses yield similar results (and in fact are on the line of unity), we therefore believe that our main inference of not finding an effect of applying the VPM in our study domain is unlikely to be fully explained due to non-adherence to our protocol. This issue is now discussed in our discussion section of our manuscript, reading as follows (changed, using track changes):*

*‘Second – as expected in this practice-based open-label pragmatic trial using an assistive risk-based treatment strategy – not all patients followed our study protocol or were able to comply to the treatment recommendation of the Vienna prediction model. Similarly, also some hospital specialists caring for patients in our control group may have been influenced due to trial participation of their patients. Nevertheless, they did not receive any information on the result of D-dimer testing. Yet obviously, both issues may dilute any true effect that would be observed should all patients and physicians have been ‘forced’ to be compliant to our risk tailored approach. Therefore, we performed secondary analyses, yielding similar results. For these secondary analyses we now used a propensity model based approach, comparing patients (between index and control) above the 75^th^ propensity quartile of being adherent. Albeit clinically an intuitive approach, other more advanced statistical approaches exist, likely inverse probability weighting. We additionally used these approaches as well, yielding very similar inferences (data not shown).’*

**7. I am confused by the fact that the predictive performance analysis excluded patients who continued anticoagulant treatment. In patients randomised to the index group, only those at higher risk (according to the Vienna prediction model itself) would have continued anticoagulant treatment. Therefore, excluding these patients limits the validation exercise to only those at lower risk. Please confirm/clarify.**

*Answer: This is an excellent question. Indeed, we now analysed the predictive performance (discrimination and calibration) only in patients that did not prolong anticoagulant treatment, i.e. in 377 patients from the control arm of our trial and 317 patients from the index arm (total 694 patients). This was done deliberately as prolonged anticoagulant treatment (obviously) lowers the risk of recurrent VTE, thereby leading to an apparent (and biased) overestimation of the predicted risk by the Vienna prediction model (VPM). Nevertheless, we also agree that excluding patients that continue anticoagulation altogether may lead to a scenario where we only validate the VPM in a lower risk population.*

*Therefore, prompted by this remark, we now added several additional analyses. First, we repeated all analyses on the predictive performance of VPM in all patients, thus also including those having received anticoagulant treatment. Results of calibration plots are shown below, showing similar results as our initial analysis including only patients not receiving anticoagulant treatment. Discrimination results of including only non-treated patients was 0.76 (95% CI 0.69-0.83) versus 0.69 (95% CI 0.62-0.77) when using all available patient information (including treated patients).*

*However, clearly, the VPM still underestimates risk in patients with a high predicted risk. We believe this further strengthens our observation that VPM likely underestimates risk in high risk groups. High risk patients are more likely to receive treatment and thus including these patients in our analyses would lower the observed risk in this risk category potentially leading to an underestimation of risk, whereas we still observe an overestimated risk.*

*Next, we performed a more advanced approach to better account for any bias induced by excluding patients on prolonged anticoagulant treatment. Hereto, we used the relatively recently developed methods as proposed by Pajouheshnia and co-workers (see BMC medical research methodology; 2017 Dec;17(1):103). This method uses inverse probability weighting (IPW). We first fitted a propensity score model (psm) regressing the likelihood of receiving prolonged anticoagulant treatment (dependent variable) using the predicted risk of recurrent VTE from the VPM as independent variable. Next, all analyses in the non-treated population are repeated, yet each non-treated patient receives a weight, based upon the inverse of the propensity of being treated, in fact: 1/(1-psm). As such, non-treated patients with a higher likelihood (propensity) of prolonging anticoagulant treatment (thus more likely the higher risk patients) receive more weight in these analyses, thereby accounting for the rightfully mentioned observation from the reviewer of only using low-risk patients in our validation analyses. The inferences of these analyses are also similar to our initial analyses, still showing underestimated risks notably for those with higher predicted risks; see below.*

*In terms of discrimination, this analysis indeed showed somewhat better performance of the VPM (c-statistic 0.80, 95% CI 0.75-0.84), yet this would in our view not strongly influence our inferences.*

*To discuss these issues in more detail, we now added the following paragraph to the limitation section of our manuscript:*

*‘Finally, our analyses on the predictive performance of the Vienna prediction model were restricted to only those not receiving anticoagulant treatment (n=694), thus in relatively more low-risk patients (as higher risk patients were more likely to receive prolonged anticoagulant treatment). Therefore, to explore the potential influence of this approach on the predictive performance of the Vienna prediction model, we performed several sensitivity analyses, e.g. including both treated and non-treated patients as well as using inverse probability weighting to account for treatment effects of anticoagulant treatment. Nevertheless, these analyses yielded similar results, strengthening our inferences (data not shown).’*

**8. Please clarify why 12 months of follow-up were selected for the validation when the primary outcome of the trial was measured at 24 months.**

*Answer: This is a valid point. To be completely clear here; in the index arm of this trial the Vienna prediction model was used to estimate the risk of recurrent VTE, where patients with an annualized (thus at 12 months) predicted risk of 5% or higher being recommended to prolong anticoagulant treatment. Patients with a predicted risk below this threshold were recommended to stop anticoagulant treatment. In the control arm, the Vienna prediction model was not used. All patients were subsequently followed-up for 24 months (see also our answer to question 3). For the validation part of our manuscript we thus used precisely the same time frame as used in the intervention strategy, which is the risk of recurrent VTE at 12 months. However, we did report the observed rate of recurrent VTE during 24 months of follow-up as well, stratified for different risk strata of predicted risk (see Figure 4). We now explained this in more detail in the section on ‘statistical analyses and power calculation’, more precisely the paragraph on the validation of the Vienna prediction model, using track changes.*

**9. The authors (lines 231-232) mention using the baseline hazard function from the original publication. Does this imply that the risk score used in the validation is different to the risk score used in the randomised trial? If so, please clarify the reason for choosing different equations.**

*Answer: Good point, and we apologize for the unclarity here as indeed, we used exactly the same baseline hazard for the randomized part of our trial and the validation part of our manuscript. This in fact is also the same baseline hazard that was derived from the development paper. See also our answer to the previous question.*

**10. Figure 2: It is difficult to see the proportions in white font over the bubbles even after downloading the original .tiff version. Please consider using a different font and/or colour.**

*Answer: We agree, we now simplified figure 2, and added information on the values of the y-axis. The full manuscript now includes 5 figures. We would like to ask the editors for advice whether or not we want to move 1 or 2 figures to the online supplementary information. We believe now the most important figures are figure 1 (flow of patients in the study), figure 4 (KM curves) and figure 5 (see below, added prompted by questions 1 and 2 from reviewer #3).*

**11. The 'validation' analysis suggest that the authors were able to calculate the risk from the Vienna prediction model in both the index and control group. Please consider reporting the number and proportion at high risk. This could potentially be added to Table 1.**

*Answer: Good point, we would like to refer to our answers to questions 1 and 2 from reviewer #3, where we describe in detail the proportions of risk strata as well as potential consequences of whether or not to prolong anticoagulant treatment based upon this estimated risk threshold.*

**12. I would suggest moving Table 2 (Description of 5 patients with interval recurrent venous thrombo-embolic event) to the online supplement as it does not appear essential to the main 'story'.**

*Answer: This has now been altered accordingly, using track changes.*

**13. The authors state that "the model underestimated recurrence risk around and above the threshold of 5%" (see lines 318-319); however, according to Figure 3 (right panel) the predicted line seems very close to every observed estimate (triangles) and certainly well within the confidence bands. Please clarify.**

*Answer: We agree, the uncertainty and 95% confidence bands are relatively wide here. Our interpretation of the data is that in patients with a lower predicted risk of recurrent VTE, the observed rates of recurrence are ‘in line’ and clinically acceptable. This is for instance best exemplified by the observed recurrence rate of 2.5% (95% CI 0.7-4.3) in patients with a predicted risk between 2% and 4% (mean predicted risk in this group 3.1%). Similarly, in those with a predicted risk between 4% and 5% the actual observed risk of recurrent VTE is 6.7% (95% 2.5-10.9), which we believe may be considered too high. We tried to clarify this more in the section on predictive performance of the VPM, using track changes, also highlight the uncertainty here.*

**14. Pending clarification of the above comment, could it be that the 'apparent' underestimation around the 5% threshold is linked to the fact that some (most?) patients with a risk above 5% continued treatment and were therefore excluded from the validation exercise. To address this concern, I would encourage the authors to consider repeating the validation analysis without excluding any patient (i.e. regardless of whether they continued treatment) as a sensitivity analysis.**

*Answer: We thank the reviewer for these comments; they prompted us to do some additional analyses, which showed similar inferences, also regarding the estimated risks around the 5%. We would like to refer to our answer to question 7 from this reviewer for further clarification.*

**15. I believe that the data should be made publicly accessible instead of available on request as per PLOS Medicine policy but will defer to the editor's guidance on this aspect.**

*Answer: We would like to refer to the first question from the editors, also relating to this topic.*


***Reviewer #2:***

The manuscript by Geersing et al. describes the results from a randomized trial conducted with the aim of comparing decision making of treatment duration of oral anticoagulant treatment after venous thromboembolism (VTE) by use of the VIENNA prediction model (VPM). The overall hypothesis was lower recurrence risk among patients assessed using a risk-based strategy (VPM) than recurrence risk found in a control group. In the VPM arm, a total of 441 was enrolled and 442 VTE patients were randomized to usual care. The duration of follow-up was 24 months.

The study was an investigator-initiator trial conducted on multiple sites with block-randomizing (site and type of VTE) of patients to either VPM or usual care. The trial followed principles of a pragmatic trial with no blinding. A CONSORT description was available and the trial was registered in a Dutch trial database. Participants presented with VTE and were eligible if no so-called 'provoking' risk factors were prevalent including active cancer. Indications for long-term treatment with an oral anticoagulant treatment was also an exclusion criteria.

Initially all patients were treated with warfarin 3 to 6 months after low molecular weight heparin lead-in. Before end of this treatment period, patients were randomized to either usual care (discretion of the physician) or to a risk-tailored approach using VPM. Extended (i.e. longer duration of the initial three to six months) duration of treatment was offered to those with a predicted risk of 5% per year or above in accordance with the ISTH recommendations. Model risk assessment was done twice, first time short before treatment cessation and second time approximately one month after. In the control group, the treatment cessation time was based on the discretion of the physician. Patients were followed up at 3, 12, and 24 months.

**Major comments:**

**1. The current clinical equipoise relates to when oral anticoagulant treatment can safely be withheld in patients presenting with VTE and no apparent clinical risk factors. Anticoagulation is effective in preventing VTE recurrence but the use of oral anticoagulant treatment has an inheriting risk of bleeding. Hence, the duration of treatment should be based on an assessment of the net clinical benefit. Therefore, it is important to present the proportion of patients (stratified according to randomization) with treatment duration in e.g. quartiles, which has been the traditional length of treatment in VTE patients (3, 6, 9, or 12 months). This would allow the reader some insights into the initial decision making that was left to the discretion of the treating physician. Preferably, this could also be subcategorized into patients presenting with DVT or PE.**

*Answer: We fully agree, this is an excellent suggestion to try to explore better where the clinical trade-off in terms of net clinical benefit (recurrent VTE versus major bleeding) would lie, depending of the threshold above which one may decide to prolong anticoagulant treatment. We would like to refer to our extensive answer to questions 1 and 2 from reviewer #3, who raised similar concerns.*

**2. It is not entirely clear when the randomization occurred. Since this was a pragmatic (non-blinded) study design, it could be hypothesized that knowing the assignment of the individual treatment arm would affect the behaviour (and preferences) of the patients and/or physicians. The authors are encouraged to reflect on the consequences of using a no-blinded study design, and on the direction and magnitude of this potential bias on the effect estimates. Furthermore, the Vienna model consists of three parameters: the extent of VTE, patient sex, and D-dimer concentration. This is obvious information, also for physicians determining anticoagulant treatment duration without VPM. In consequence, this is likely to result in no difference between the two treatment arms. This dilemma could be discussed in the article.**

*Answer: Good points. We first of all would like to refer to our answer to question 3 from reviewer #1, who also asked of our randomization strategy. We agree with the reviewer that indeed hospital specialists caring for patients in our control group could potentially be influenced due to trial participation of their patients. Hospital specialists however were not actively involved in this trial, nor did they receive e.g. information on the results of D-dimer testing in the control arm. As such, we anticipate that this influence is likely very small. Nevertheless, we fully agree that this dilemma needs further discussion, and we now added it to our ‘strengths and limitations’ section of our manuscript, using track changes. Furthermore, we believe this question at least in part also relates to the next question of secondary analyses, and we would like to refer to our answer there as well.* 

**3. The secondary analysis, which was not pre-planned, is of interest as these estimates may reflect a more realistic effect of what would be seen in everyday clinical practice. However, the implementation of the approach is lacking details. The authors are encouraged to consult this reference DOI: 10.1056/NEJMsm1605385, which outlines an appropriate analytic plan to conduct per-protocol analyses specifically for pragmatic trials that minimizes the risk of selection bias in relation to censoring. Additionally, the following sentence is unclear: (p7) "we used patients within the 75th quartile of predicted probabilities of protocol adherence." Was the data restricted based on the propensity score? If so, it would be important to characterize the patients who did not enter this analysis - what were the main differences in terms of baseline characteristics? Perhaps an approach like inverse probability of treatment (or rather protocol adherence) weighting could allow for a more
efficient use of the available data?**

*Answer: These are excellent questions. We indeed also performed an IPW analysis, yielding similar results. We would also like to refer to our extensive answer to question 6 from reviewer #1, who raised similar concerns.*

**4. What is the argument for assessing performance only among patients who did not receive prolonged treatment? This would be a selected group of patients with a presumed low recurrence risk. Calibration in high risk patients would be of clinical relevance.**

*Answer: This is good and valid point, also raised by reviewer #1. We would like to refer to our answers to questions 7 and 13 from this reviewer.* 

**5. The performance, in terms of calibration, of VPM is assessed using 4 probability categories (0-2%; 2-4%; 4-5%; and >5%). However, the clinical relevant cut off point is at 5%, as stated by the International Society of Thrombosis and Haemostasis. At this threshold the VPM is not well calibrated. The authors should deliberate on the consequence of bad calibration at this clinically meaningful cut-off point.**

*Answer: We agree that this precisely may be the topic warranting future clinical debate. We expect that likely this current threshold of 5% will be lowered in future ISTH communications, also and perhaps in particular due to the fact that recent NOAC extension trials showed a residual VTE recurrence risk of around 2% per year. Yet, that would imply prolonging anticoagulant treatment in all patients, which we believe may be undesirable as well because of the associated bleeding risk in particular ‘in the long run’. Thus, maybe the future threshold is more likely to be around 3% to 4%, thereby still allowing a substantial population to stop treatment after the initial treatment phase. We deliberated more of these issues in our section of clinical implications, and improved it accordingly (using track changes). We would also like to refer to our answer to questions 1 and 2 from reviewer #3, who raised similar concerns.* **6. It would be relevant to include relative estimates with 95% CI between treatment arms on risk of bleeding.**

*Answer: This was added, using track changes.*

**Minor comments:**

**7. Could the authors please check the order of the figures/tables in the supplemental material?**

*Answer: Supplementary material was checked and extended following a few additional analyses, using track changes.*

**8. Please provide labels on the y-axis for Figure 2.**

*Answer: We would like to refer to our answer to question 10 from reviewer #1 who also had difficulty with reading figure 2. It has now been changed and simplified accordingly.*

**9. What is meant with the term "impact study" in the title? Consider to leave it out.**

*Answer: The editors also suggested changing the title. We now deleted the word impact. The full title now is: ‘Effect of tailoring anticoagulant treatment duration by applying a recurrence risk prediction model in patients with venous thrombo-embolism compared to usual care: a randomized controlled trial.’ The short title now is: ‘Clinical effects of estimating recurrence risk in VTE patients.’*

**10. The study excludes patients with a history of VTE within 10 years. Yet, table 1 list patients with a "history of DVT or PE". Please clarify, if this is a VTE more than 10 years ago? (and supplementary table S1).**

*Answer: Yes, indeed, this is VTE with a history of DVT or PE longer than 10 years ago. This is now clarified in the footnotes of table 1, using track changes.*

**11. Table 1: proportion of patients receiving hormonal therapy - please clarify if this proportion is for women only. (and supplementary table S1).**

*Answer: Yes, indeed, this is in women only, also clarified in the footnotes of table 1, using track changes.*

**12. It could be mentioned (and discussed) that VPM has previously been externally validated.**

*Answer:* *The reviewer is likely referring to the following publication: ‘Risk of Recurrence After a First Unprovoked Venous Thromboembolism: External Validation of the Vienna Prediction Model With Pooled Individual Patient Data; J Thromb Haemost 13 (5), 775-81 May 2015’. It is indeed a good suggestion to mention this external validation study also in our main manuscript, particularly as similar to our inferences it showed that the VPM tends to underestimate recurrence risk. We now discussed and added this reference to our paragraph of ‘comparison with existing literature’ in the discussion of our manuscript, using track changes.*

**13. It could be highlighted that the authors are not identical with the ones developing the VPM.**

*Answer: Indeed, it may be important to acknowledge this. To clarify this, we now added in the introduction that we validated VPM in a different setting and country as compared to where the VPM was derived (i.e. Austria).*

**14. Table 1: "Index" could be rephrased to "VPM patients".**

*Answer: This has been changed accordingly, using track changes.*

**15. Figure 1: 10 patients are excluded because of clear indication for short treatment. Why and what is this?**

*Answer: We can understand the unclarity here. In fact, a total of 50 patients withdrew informed consent (these are mentioned in figure 1 as ‘patient or physician wish against randomisation’, 25 patients in each group). Additionally, 10 patients were not randomized and thus not included in our analyses because the treating physician was absolutely against treating the patient for a prolonged time with anticoagulation, mainly because he/she deemed the VTE event as provoked rather than unprovoked. This sometimes can be unclear, see also our answer to question 4 from reviewer #3.*

***Reviewer #3:***

In this randomized controlled trial, the Vienna Prediction Model (VPM) was tested to determine whether implementing the model would reduce recurrence risk. 883 patients were randomised to using the VPM compared with usual care. No difference in number of recurrent VTE events was observed, and slightly more CRNM bleeding in the intervention arm.  The authors conclude that application of the VPM in patients with unprovoked VTE is unlikely to reduce overall recurrence risk.

This is a well-described, honest and important account of how a promising prediction model can fail to translate in improved clinical care, and therefore imperative to be noticed by both clinicians and researchers. For this reason it is also, possibly even more so than for 'positive' studies, necessary to describe in detail what happened.

*Answer: We thank the reviewer for his/her positive remarks on our work.*

**Most important comments:**

**1. This study in fact investigates 2 things at the same time: the performance of the VPM and the related antithrombotic strategy. The authors now conclude that the absence of a difference in risk is related to the application of the model. However, it could also simply be due to insufficient treatment related to the result of the model. Theoretically the recurrence risk in both study arms should be equal. So, to find a difference in actual VTE occurrence, a strategy should have been in place that would strongly impact this risk. This has not really happened, so it seems, considering that the rate of prolonged treatment was in fact low in both arms, although a bit higher in the intervention arm: about 25 vs 10% (if I infer correctly from fig 2). So all effect from applying the model should have come from this small difference in treated subjects, which seems implausible. This option, of an insufficient treatment difference between the arms, could receive more attention in the discussion.**

**2. In fact I think it could be modelled (e.g. as in a Mendelian Randomisation analysis) how many more cases could have been prevented if the difference should have been larger between the groups (and likewise the bleedings). This would clearly not be the same as in a true RCT but it might shed some light on the respective role of the VPM and the applied treatment strategy in the observed results of this study.**

*Answer: We would like to thank the reviewer for these questions as they are highly relevant. They strongly relate to each other, therefore, we will answer them together. Indeed, as also mentioned by reviewer 2, the current clinical practice in the DOAC-era is that patients with unprovoked VTE are almost always offered the option (or even recommendation) to prolong anticoagulation to minimize their risk of recurrent VTE. Paradoxically, this recommendation is even stronger because validated tools to predict the risk of recurrence are not available, notwithstanding the fact that we also know that recurrence risk is mixed in the population of patients with unprovoked VTE. So, in terms of clinical implications, we believe it indeed would be informative to explore what could have happened in our trial in the current antithrombotic landscape of prolonging (DOAC) treatment in most patients.*

*Hereto, as suggested by the reviewer, we tried to model several scenario’s in a hypothetical cohort of 1000 patients with unprovoked VTE, followed-up for 5 years. First, based upon our own observations, we know that the proportion of patients categorized in the following risk strata of annualized VTE recurrence risk of 0-2%, 2-4%, 4-5%, or >5% are 4.9%, 35.1%, 17.1%, and 42.9%, thus 49, 351, 171, and 429 patients in a hypothetical cohort of 1000 patients, respectively. Based upon our VISTA data, we also know the observed annualized rates of recurrent VTE in these risk strata if anticoagulant treatment is withheld, i.e. 0.0%, 2.5%, 6.7%, and 14.4%, respectively. We know from the NOAC extension trials that these risks of recurrent VTE can be lowered with an observed hazard ratio in these trials of (about) 0.25, thus yielding annualized rates of recurrent VTE if managed with prolonged NOAC-treatment to be 0.0%, 0.63%, 1.68%, and 3.6%, respectively. Now, let’s assume the annualized rates of recurrent VTE to be constant over a 5-year period. This is a conservative assumption, as we know that the rates of recurrent VTE are highest in the first year after the initial event and typically a little bit lower in the subsequent years. Using these data, we can estimate the number of VTE events over a 5 year period in our hypothetical cohort of 1000 VTE patients for the following scenario: (i) prolong treatment in all patients, (ii) prolong only if the estimated risk of recurrence is >2%, (iii) prolong only if the estimated risk of recurrence is >4%, (iv) prolong only if the estimated risk of recurrence is >5%, or (v) do not prolong anticoagulant treatment altogether. In fact, the number of recurrent VTE events for these scenario’s would be 103, 103, 135, 178, and 410 events; or compared to prolonging treatment in all patients (first scenario): 0, 0, 33, 76, and 308 additional events, respectively.*

*Similarly, we can estimate the number of major bleeding events induced in these different scenario’s. Unfortunately, the annualized rates of major bleeding in VTE patients on prolonged DOAC-treatment is less certain, so let’s assume the number of major bleeding events for three different annualized rates of major bleeding: 0.5%/year, 1.0%/year, and 1.5%/year. Over a 5-year period, we can then calculate the number of major bleeding events for these different rates of major bleeding. All these numbers are depicted in the following table, that is now also included as a Supplementary Table S3.*

**Supplementary Table S3: Clinical outcomes in a hypothetical cohort of 1000 VTE patients, followed-up for 5 years**

| **Treatment strategy** | **Number of treated patients** | **Recurrent VTE** |  | **Major bleeding** |  |
| --- | --- | --- | --- | --- | --- |
|  |  | *number* | *additional VTE* |  | *number* |
| **Treat all patients** | 1000 | 103 | 0 | Rate 0.5% per year | 25 |
|  |  |  |  | Rate 1.0% per year | 50 |
|  |  |  |  | Rate 1.5% per year | 75 |
| **Treat only >2% risk/year** | 950 | 103 | 0 | Rate 0.5% per year | 24 |
|  |  |  |  | Rate 1.0% per year | 47 |
|  |  |  |  | Rate 1.5% per year | 71 |
| **Treat only >4% risk/year** | 600 | 135 | 33 | Rate 0.5% per year | 13 |
|  |  |  |  | Rate 1.0% per year | 27 |
|  |  |  |  | Rate 1.5% per year | 40 |
| **Treat only >5% risk/year** | 430 | 178 | 76 | Rate 0.5% per year | 9 |
|  |  |  |  | Rate 1.0% per year | 17 |
|  |  |  |  | Rate 1.5% per year | 26 |
| **Treat no patients** | 0 | 410 | 308 | Rate 0.5% per year | 0 |
|  |  |  |  | Rate 1.0% per year | 0 |
|  |  |  |  | Rate 1.5% per year | 0 |

*Subsequently, we can plot these numbers graphically with on the x-axis the threshold above which prolonged anticoagulant treatment is prescribed, and on the y-axis the number of additional VTE events and major bleeding events (for three different rates of major bleeding) induced by these different strategies. This graphical plot would then be as follows:*

*This graphical plot illustrates that indeed somewhere between the threshold of 3% and 4% the number of additional VTE events induced by treating less patients with unprovoked VTE equals the number of major bleeding events induced due to prolonged NOAC-treatment.*

*We now added this Figure to the main body of manuscript, and updated our section on clinical implications describing these issues, using track changes. It now reads as follows:*

*‘Clinical applicability and research implications*

*Given our inability to demonstrate a reduction on recurrence risk by risk-stratifying care and in light with the publication of recent extended NOAC-trials that showed superiority of extended NOAC-treatment over either placebo or aspirin for reducing recurrence risk in patients with (mainly) unprovoked VTE, it may be tempting to consider prolonging anticoagulant treatment in all patients with unprovoked VTE, thus without any method of assessing recurrence risk. Indeed, in these trials extended NOAC-treatment is associated with a hazard ratio for recurrent VTE (compared to placebo) of around 0.25 and a substantially lower residual recurrence risk of around 1.5 to 2 % per year at an acceptable low rate of major bleeding (around 0.5% per year). Nevertheless, in these trials, follow-up duration is still limited, (frail) elderly were underrepresented, as were patients with a high bleeding risk, all limiting generalizability to real-life clinical care. Moreover, our findings do indicate that in a large proportion of patients (nearly half of the population included in our trial) a low predicted risk (up to an annualized rate of 4%) is correlated with a similarly low observed risk, indicating that they in fact could safely stop anticoagulant treatment based upon a formally estimated risk rather than an ‘educated guess’ which is likely more influenced by clinical experience and setting. The threshold on what then defines ‘low VTE recurrence risk’, or what residual recurrence risk is (still) acceptable, is open for clinical debate. For instance, in a hypothetical cohort of 1,000 unprovoked VTE patients, 950, 600, and 430 patients will be classified as having an annualized recurrence risk >2% (low risk), >4% (moderate risk), or >5% (high risk), respectively, based upon VISTA observations. Using the observed rates of recurrence from VISTA for these strata, as well as the known hazard ratio of 0.25 for prolonged anticoagulant treatment (compared to placebo), we can estimate the number of recurrent VTE events for different treatment strategies over a time period of 5 years. Similarly, this can be done for the number of bleeding events. The rate of major bleeding for prolonged NOAC treatment is less well-defined in the literature, so let’s assume three different major bleeding rates (0.5%/year, 1.0%/year, and 1.5%/year). See Supplementary Table S3 for further explanation. Next, we can plot the number of additional VTE and major bleeding events induced due to prolonging anticoagulant treatment (y-axis) for certain risk threshold above which patients are recommended to prolong treatment (x-axis). In Fig 5, it may be appreciated that this threshold should perhaps be somewhere around 3% to 4%, as then the number of additional VTE events induced because we treat fewer patients are more or less similar to the number of bleeding events that occur given that antithrombotic treatment strategy. This thus is lower than the 5%, which is currently (still) the recommend threshold of ‘low-risk’ as defined by ISTH standards. Also, we should acknowledge that in particular at this threshold of 5% the Vienna prediction model is less well calibrated, further exemplifying that perhaps a threshold of 3% or 4% is more likely to be clinically meaningful.’*

**3. Why was the rate of treatment so low anyway? Was only such a small proportion of patients classified above 5% per year? I find this information not easy to find in the manuscript: which patients took OAC or not at what predicted risks and what were their observed risks, with and without treatment. This would also give some information on effectiveness of the treatment given equal estimated risks. It could also be discussed how the low treatment rate relates to the current guidelines in which everybody with an unprovoked first event is recommended to continue unless a high bleeding risk is present.**

*Answer: We believe part of this question is answered in our descriptions and answers on the two first questions as well. Using the known hazard ratio of preventing recurrent VTE (see our answer to the previous two questions), we can model the observed rates of recurrent VTE for different risk strata, as also explained above. To be fully transparent, these rates would be as follows:*

|  | *Proportion of patients* | *Non-treated, annualized risk* | *Treated, annualized risk* |
| --- | --- | --- | --- |
| *Risk between 0-2%* | *4.9%* | *0.00%* | *0.00%* |
| *Risk between 2-4%* | *35.1%* | *2.50%* | *0.63%* |
| *Risk between 4-5%* | *17.1%* | *6.70%* | *1.68%* |
| *Risk above 5%* | *42.9%* | *14.4%* | *3.60%* |

*We believe this information is now also essentially captured in Supplementary Table S3, but can also be added as an additional Supplementary Table, if deemed appropriate by the editors.* 

**Other comments:**

**4. Please mention how the criteria used for defining an unprovoked event relate to other definitions (from other scores or guidelines, such as the ISTH's).**

*Answer: Excellent question. In fact, during the conduct of the VISTA trial, the ISTH issued the following definition of unprovoked VTE (in 2016, co-authored by one of the authors of this manuscript, GJG, see ‘Categorization of patients as having provoked or unprovoked venous thromboembolism: guidance from the SSC of ISTH’ JTH; Volume 14, issue 7, July 2016);*

*‘VTE is defined unprovoked in the absence of a (major or minor) transient or persistent risk factor. Major transient risk factors include recent surgery or confined to a hospital bed for more than 3 days; minor transient risk factors include e.g. oestrogen use, but also surgery for less than 30 minutes or confined to a bed for more than 3 days out of the hospital setting; finally persistent risk factors include notable an active malignancy.’*

*We defined unprovoked as having no previous surgery (i.e. lasting longer than 30 minutes under general or spinal anaesthesia) or casting after lower-limb injury within 3 months prior to the VTE event, or pregnancy/puerperium up to 6-weeks post-partum. In contrast to current ISTH definitions, we did allow oral contraceptive related VTE in female patients to be included in our trial, given that at the time of VISTA inception there was much uncertainty whether these events need to be classified as provoked or unprovoked. Patients with a history of VTE within the past 10 years were excluded, as were patients with an active malignancy (i.e. latest treatment less than 6 months prior to the VTE event or in palliative care). In both patient groups, there is not much discussion that recurrence risk is high warranting prolonged anticoagulant treatment.*

*Hence, notably the inclusion of oral contraceptive related VTE in our trial is an important difference between our definitions of unprovoked and current ISTH definitions. We discussed this issue now in more detail in the section of strengths and limitations, using track changes.*

**5. Risk estimation was performed twice, namely shortly before treatment cessation at the end of the initial treatment period (thus while still using anticoagulant treatment) and approximately one month after treatment cessation" This is rather confusing for both patients and doctors? Could this have affected the results and would this strategy impede implementation of the model?**

*Answer: Agree, this is done as D-dimer levels can be influenced by anticoagulation use, i.e. they are lowered by the use of anticoagulation. During the performance of the trial this was not in particular confusing. Rather, we explained that after the initial treatment phase (3-6 months), we would – only for a few weeks – let the anticoagulation system ‘free from anticoagulation control’ to see what would happen. We then could easily explain to patients that if D-dimer levels then increased this was a clear indication that in relative short notice the anticoagulation cascade was becoming activated again, thus possibly warranting reinitiating anticoagulant treatment. The biggest concern with this approach is that in very high risk patients we would already observe a recurrent VTE event during these first weeks. In fact, this happened in 5 patients. These patients are now described in Supplementary Table S2.*

**6. How were possible recurrent events adjudicated as such?**

*Answer: We would like to refer to our answer to question 4 from reviewer #1, who had a similar remark.*

**7. Were all patients followed for 24 months or until recurrence occurred? Not clear if people were lost to follow-up?**

*Answer: No patients were lost to follow-up. In case we were unable to contact patients by phone, clinical information on the occurrence of recurrent VTE or bleeding events was obtained from their primary care doctor. Further follow-up indeed was discontinued after the occurrence of recurrent VTE. This is now made more clearly in the section on follow-up appointments, using track changes.*

**8. 46 patients in the index group did not obtain any risk estimation whereas an additional 58 patients (total of 104) were for a variety of reasons unable to comply to the treatment recommendation" It is unclear which 58 patients is referred to from fig 1.**

*Answer: Indeed, we can understand this question. We ‘struggled’ with this ourselves as well (how to present the flow of patients). In fact, Fig.1 shows us the actual received treatment after each risk estimation. This is not by definition the same as the recommended treatment. The 58 patients notable refer to patients that stopped anticoagulant treatment after VPM 1 or VPM 2, even though they were recommended to continue. So in total 104 patients were ‘non-compliant’ to the VPM recommendation: in 46 patients they didn’t show up for the risk estimation at all, and in 58 patients they did went for D-dimer testing in order to calculate their recurrence risk, but they did not comply with the treatment recommendation, notably stop anticoagulant treatment whereas the recommendation was to continue.*
